# The Underlying Pharmacological Mechanisms and Active Components of XZZTP in Modulating Bacterial Inflammation Elucidated by LC-MS/MS, Network Pharmacology, In Vitro Experiments, Molecular Docking, and Dynamics Simulations

**DOI:** 10.3390/ph19050678

**Published:** 2026-04-27

**Authors:** Qianli Kang, Fangyuan Deng, Sen Li, Ting Wang, Hongmei Lin

**Affiliations:** 1 College of Chinese Materia Medica, Beijing University of Chinese Medicine, Beijing 100029, China; 20220941474@bucm.edu.cn (Q.K.); 20230935174@bucm.edu.cn (F.D.); 17832049290@163.com (S.L.); 2 Key Laboratory of Traditional Chinese Medicine Research and Evaluation, National Medical Products Administration, Beijing 100029, China; 3 Beijing Academy of Chinese Medicine, Beijing University of Chinese Medicine, Beijing 100029, China; 4 Key Laboratory of Famous Doctors’ Classic Prescriptions, National Administration of Traditional Chinese Medicine, Beijing 100029, China; 5 Center for New Drug Research and Development of Chinese Materia Medica, Beijing Academy of Chinese Medicine, Beijing University of Chinese Medicine, Beijing 100029, China

**Keywords:** Xiao Zhong Zhi Tong Patch, LC-MS/MS, Franz diffusion, bacterial inflammation, network pharmacology, PI3K/AKT/HIF-1α pathway, molecular docking, molecular dynamics simulation

## Abstract

**Background**: The Xiao Zhong Zhi Tong Patch (XZZTP) has been extensively utilized in China to alleviate many diseases associated with bacterial inflammation. However, its pharmacological mechanism and active components remain unclear. **Methods**: The anti-inflammatory effects of XZZTP were evaluated in vivo and in vitro models. The characterization of XZZTP and its transdermal components was performed using LC-MS/MS. The underlying pharmacological mechanism was predicted through network pharmacology using the identified transdermal components and verified by Western blotting. Molecular docking and molecular dynamics simulations were performed on key targets to screen active components. **Results**: XZZTP showed a swelling inhibition rate of 45.96% in xylene-induced ear edema mice in vivo. In vitro, the inflammatory mediators NO, TNF-α, and PGE2 were concentration-dependently reduced by XZZTP in the LPS-induced RAW 264.7 macrophages model, with inhibition rates of 56.53%, 53.75%, and 48.49% at 200 µg/mL, respectively. LC-MS/MS identified 126 chemical components (97 newly reported) in XZZTP, including 52 transdermal potential active components, among which a new iridoid and its isomer were reported for the first time. Network pharmacology analysis demonstrated that XZZTP mainly downregulated the PI3K/AKT/HIF-1 signaling pathway to alleviate bacterial inflammation. The protein expression of core targets p-PI3K, p-AKT, and HIF-1α in the LPS-induced RAW 264.7 macrophages was significantly reduced after XZZTP intervention. Eight active components were screened via molecular docking, and molecular dynamics simulations of three representative complexes validated stable binding interactions, supporting their therapeutic potential. **Conclusions**: These findings provide a theoretical basis for XZZTP as a potential agent to ameliorate bacterial inflammation-related diseases, serving as a reference for its further application.

## 1. Introduction

Bacterial inflammation is the underlying cause of many common infectious diseases, posing a severe threat to global public health, with a devastating impact on regions lacking adequate hygiene and robust public health systems [1]. Based on whether they are the primary pathogens, these diseases can be categorized into those directly caused by bacterial infection, such as pneumonia, diarrhea, tonsillitis, bronchitis, and, most severely, sepsis, which has a global mortality rate of up to 19.7% [2]. Secondary bacterial infection occurs when pathogens invade and disrupt the host’s mucosal barriers, allowing bacterial colonization, following conditions such as the common cold, mumps, and fever. For instance, nearly 17% of SARS-CoV-2 infections are accompanied by co-infections with bacterial pathogens such as *Pseudomonas aeruginosa* and *Staphylococcus aureus* [3]. The main clinical approach to treat diseases caused by bacterial inflammation is antibiotic therapy. However, the primary function of antibiotics is to eliminate or inhibit bacteria, and they cannot directly and effectively alleviate the excessive or dysregulated inflammatory response triggered by bacteria. This is particularly evident in severe infections, such as sepsis, where the fatal outcomes are often closely related to the host’s uncontrolled response to infection, rather than being solely caused by the pathogen itself [4]. Finding effective drugs that can precisely regulate the host’s inflammatory response without impairing normal immune function is a key scientific challenge in the field of infectious diseases that urgently needs breakthroughs.

Characterized by its use of multi-component formulations and multi-target synergistic actions, traditional Chinese medicine (TCM) has been practiced for thousands of years. Its proven therapeutic benefits against inflammation position it as a valuable foundation for contemporary drug discovery efforts. XZZTP is a pioneering TCM product in China that is externally applied, featuring a unique, separated design of a medicinal patch and liquid. XZZTP works by clearing heat, eliminating dampness, cooling blood, detoxifying, relieving stagnation, and alleviating pain. The medicinal patch is made from dried seeds of *Artemisia halodendron* Turcz.ex Bess. or *Artemisia Sphaerocephala* Krasch. by freeze-drying (Chinese name “Sha Hao Zi”, abbreviated as SHZ). SHZ possesses the efficacy of expelling wind-dampness and drawing out pus and toxins. Its primary constituents include polysaccharides, flavonoids, phenolic acids, and volatile oils [5]. Current research has largely focused on its polysaccharide components, while studies on other chemical components remain insufficient. Pharmacological investigations have revealed that SHZ exhibits anti-inflammatory and asthma-relieving properties [6,7,8]. The medicinal liquid consists of *Pseudolysimachion linariifolium* subsp. *Dilatatum* (Chinese name “Shui Man Jing”, abbreviated as SMJ) extracts and menthol. SMJ exhibits heat-clearing, detoxifying, diuretic, cough-relieving and phlegm-dissolving properties. Its main components are iridoid glycosides, flavonoids, phenols, and volatile oils. Pharmacological studies have demonstrated that SMJ has antibacterial and anti-inflammatory activities [9,10]. Although SHZ and SMJ are widely used in folk practice, relevant scientific research is still limited.

Based on 20 years of clinical application, a method has been developed involving mixing herbal powder with different functions with the XZZTP medicinal liquid to form a paste-like substance. The paste is then applied to the XZZTP medicinal patch to treat many diseases associated with bacterial inflammation, such as diarrhea [11,12], bronchitis [13], pneumonia [14], tonsillitis [15], mastitis [16], and mesenteric lymphadenitis [17], as well as inflammatory symptoms such as fever [18] and cough [19] with different medications applied externally or/and antibiotics administered orally. Zhang et al. [20] compared the efficacy of Evodiae Fructus powder combined with XZZTP or with gauze in treating children with diarrhea externally, with rates of 96.7% and 80.0%, respectively. This difference was statistically significant (*p* < 0.05), confirming that the XZZTP can enhance the therapeutic efficacy of the combined medication. The primary role of XZZTP is to mitigate excessive inflammation induced by bacterial infection, thereby exerting a synergistic effect with other antibacterial agents to achieve therapeutic efficacy against bacterial inflammation. Above all, XZZTP pharmacodynamic effects and molecular mechanism, as well as corresponding active components still remain unclear, highlighting the urgent need for further in-depth investigation.

Building on the aforementioned research background, this study intended to conduct a comprehensive investigation of XZZTP, including pharmacological effects, pharmacological mechanisms, and active components. To verify the anti-inflammatory activity of XZZTP, the xylene-induced ear edema in vivo and the lipopolysaccharide (LPS)-induced RAW 264.7 macrophage inflammation model in vitro were established to evaluate the anti-inflammatory effects of XZZTP. LC-MS/MS coupled with in vitro Franz diffusion was utilized to characterize the full chemical components and in vitro transdermal components of XZZTP. Network pharmacology predicted the pharmacological mechanism of XZZTP in addressing bacterial inflammation and the results were verified by the protein expression levels of core targets in the key signaling pathway with Western blotting in the LPS-induced RAW 264.7 macrophage model. Finally, molecular docking was employed to screen the active components of XZZTP. Subsequent molecular dynamics(MD) simulations were performed to validate the stability of the protein–ligand complexes over time.

## 2. Results

### 2.1. In Vitro and In Vivo Anti-Inflammatory Effects

#### 2.1.1. Xylene-Induced Ear Edema in Mice

The xylene-induced inflammation model is a stable model for evaluating acute inflammation. XTTP is a patch formulation with a separately stored liquid. Similar to XZZTP, it possesses the effects of reducing swelling and relieving pain. Therefore, XTTP was employed as a positive control. In terms of alleviating ear swelling, both the positive control and XZZTP groups exhibited a significant reduction in the degree of ear swelling compared with the model group, with swelling inhibition rates of 49.93% and 45.96%, respectively (both *p* < 0.01) (Table 1). In terms of degree of ear swelling, there was no statistically significant difference in efficacy between XZZTP and the positive control XTTP.

**Figure 1:**
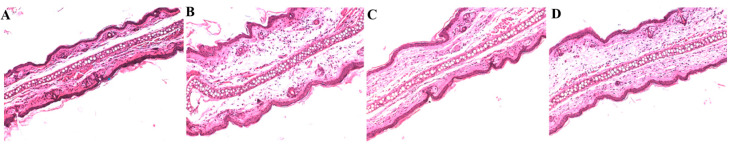
Photomicrographs of ear tissues stained with H&E: (**A**) the left ear of the model group, (**B**) the right ear of the model group, (**C**) positive control group, (**D**) XZZTP group (magnification, ×40).

The hematoxylin and eosin (H&E) staining examination of the ear sections is depicted in Figure 1. Compared with the left ear of the model group (Figure 1A), the right ear (Figure 1B) with xylene-induced inflammation exhibited a severe degree of inflammatory cell infiltration and oedema, proving the successful construction of the inflammation model. Compared with the model group, the degree of inflammatory cell infiltration and oedema in the positive control group (Figure 1C) and the XZZTP group (Figure 1D) was significantly reduced, indicating that they both have a significant anti-inflammatory effect in the xylene-induced inflammation model.

#### 2.1.2. LPS-Induced RAW 264.7 Macrophages Bacterial Inflammation Model

##### Cell Viability Results

**Figure 2:**
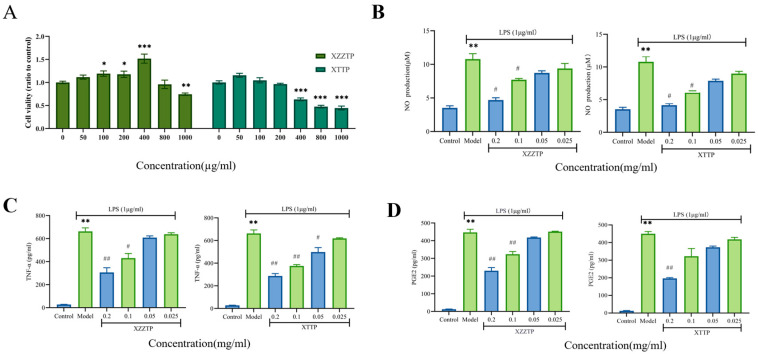
Effects of XZZTP and XTTP on cell viability: (**A**) and NO (**B**), TNF-α (**C**), PGE 2 (**D**) levels. Data are displayed as mean ± SD (*n* = 3). Compared with the control group, *** *p* < 0.001, ** *p* < 0.01, * *p* < 0.05; compared with the model group, ## *p* < 0.01, # *p* < 0.05.

The maximum non-toxic concentration of XZZTP in RAW 264.7 macrophages was determined using the CCK-8 assay. As shown in Figure 2A, the viability of RAW 264.7 cells was 95.92% after treated with XZZTP at a concentration as high as 800 μg/mL for 24 h, which showed no significant difference compared with the blank control (*p* > 0.05). In contrast, the viability of RAW 264.7 macrophages treated with XTTP at 400 μg/mL was 63.30%, demonstrating a statistically significant difference from the blank control (*p* < 0.001). Based on these findings, a concentration range of XZZTP and XTTP (25, 50, 100, and 200 μg/mL) was selected for subsequent in vitro anti-inflammatory studies.

##### Inhibitory Effects of XZZTP on TNF-α, PGE2 and NO

LPS is a pathogenic factor released by pathogenic bacteria, serving as a core pathogen-associated molecular pattern (PAMP) that triggers excessive inflammatory responses in the host [21]. It can activate macrophages to secrete proinflammatory cytokines such as nitric oxide (NO), tumor necrosis factor-α (TNF-α), and prostaglandin E2 (PGE2) [22]. Although their initial release serves to clear pathogens, excessive or sustained production exacerbates tissue damage and drives disease progression. Therefore, evaluating the effect of XZZTP on these key inflammatory mediators is crucial for assessing its therapeutic efficacy. This study established a LPS-induced RAW 264.7 macrophage model to evaluate the anti-inflammatory activity of XZZTP by detecting changes in the levels of inflammatory mediators NO, TNF-α, and PGE2.

Stimulation with LPS significantly elevated the secretion of key inflammatory mediators NO, TNF-α, and PGE2 compared to the control group (*p* < 0.01), confirming the successful establishment of the inflammatory model. Treatment with XZZTP (25, 50, 100, and 200 μg/mL) of RAW 264.7 macrophages resulted in a concentration-dependent inhibition of NO, TNF-α, and PGE2 production. At 200 μg/mL, treatment with XZZTP resulted in inhibition rates of NO, TNF-α, and PGE2 in RAW 264.7 macrophages of 56.53% (*p* < 0.05), 53.75% (*p* < 0.01), and 48.49% (*p* < 0.01), indicating a highly significant difference compared with the model group. The concentration-dependent inhibition was also observed in the positive control group treated with XTTP (Figure 2B–D). When XTTP at concentrations of 200 μg/mL, the inhibition rates of NO, TNF-α, PGE2 in RAW 264.7 macrophages were 61.52% (*p* < 0.05), 56.65% (*p* < 0.01), 56.07% (*p* < 0.01), demonstrating a statistically significant difference compared with the model group. By the way, at 200 μg/mL, no significant difference was noted between XZZTP and XTTP in the inhibition rates of NO, TNF-α, and PGE2 (*p* > 0.05).

### 2.2. Identification of Chemical Components in XZZTP

The identification of the chemical component of XZZTP is fundamental to research the underlying pharmacological effects and mechanism. The chemical components of SHZ and SMJ samples were detected using LC-MS/MS in both positive and negative ionization modes to support the comprehensive characterization of XZZTP components. A total of 67 components were identified in SHZ, including 36 phenylpropanoids, 27 flavonoids, two fatty acids, and two amino acids, while in SMJ, 59 components were identified, comprising 23 iridoids, nine flavonoids, eight phenylpropanoids, seven fatty acids, six organic acids, four saponins, and two phenethyl glucosides by combining reference standards and mass spectrometry fragmentation patterns. Notably, our LC-MS/MS analysis revealed 60 previously unreported components in SHZ and 37 in SMJ. The newly identified components in SHZ were primarily phenylpropanoids, while those in SMJ included iridoids, organic acids, saponins, and fatty acids. It is worth noting that according to the fragmentation pattern of mass spectrometry, six isomeric iridoids that have not been previously reported in the plants belonging to the families and genera of SMJ were also discovered (peaks No. 9, 10, 11, 17, 20, 22). 

**Figure 3:**
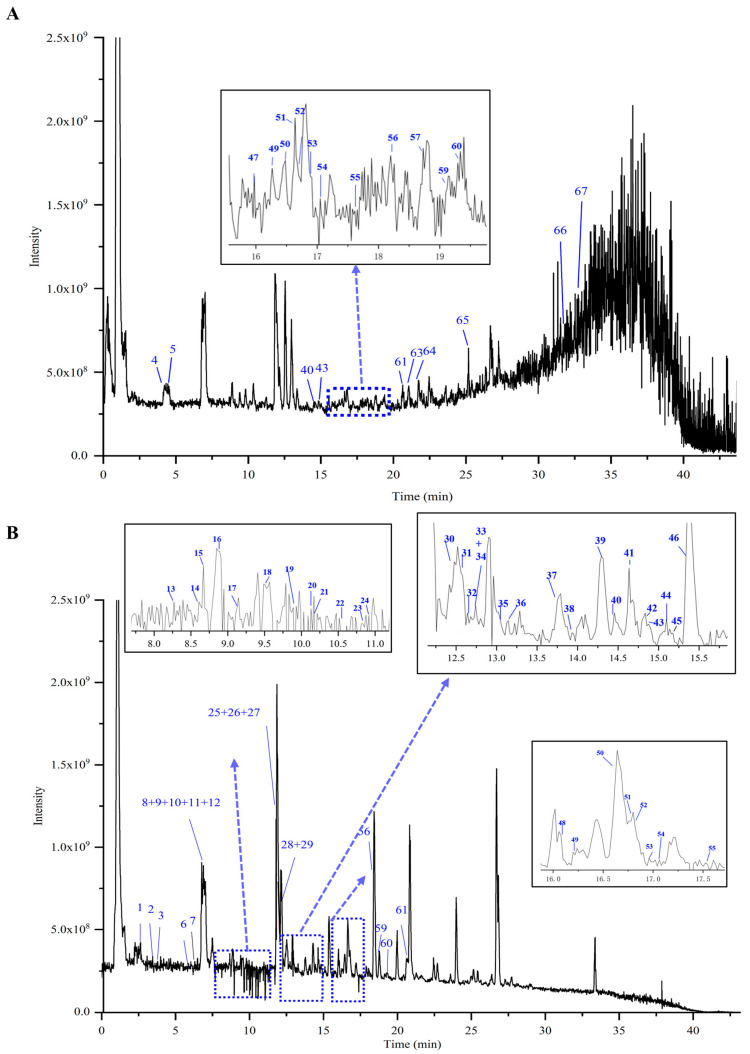
TICs for the SHZ sample in positive ion mode (**A**) and negative ion mode (**B**).

**Figure 4:**
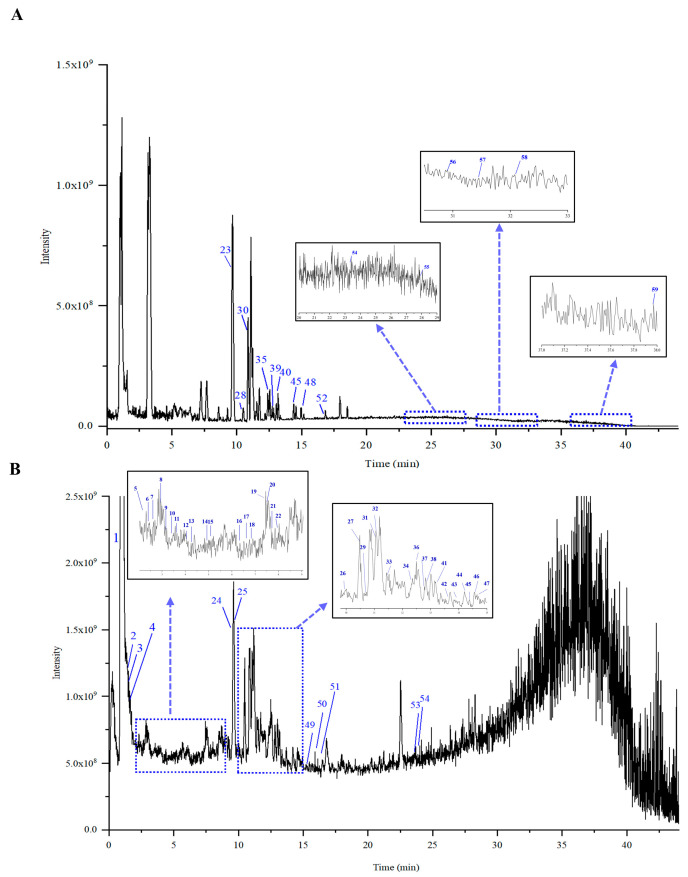
TICs of SMJ sample in positive ion mode (**A**) and negative ion mode (**B**).

**Figure 5:**
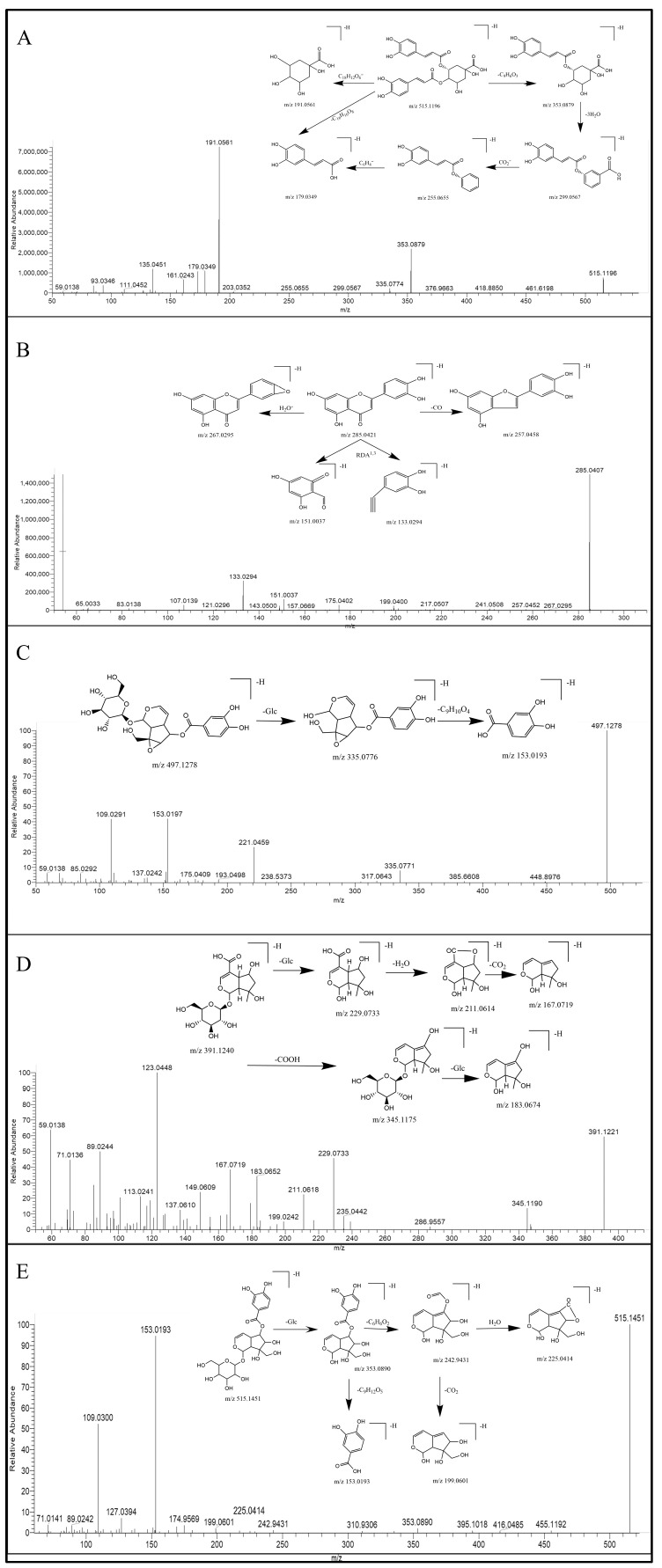
MS^2^ spectra and the corresponding proposed fragmentation. (**A**) 3,4-dicaffeoylquinic acid; (**B**) luteolin; (**C**) Verproside; (**D**) Shanzhiside (**E**); Compound-**1**.

Representative Total Ion Chromatograms (TICs) of SHZ and SMJ are shown in Figure 3 and Figure 4. Detailed information on the identified peaks, including the retention time, molecular formula, adduct ion type, detected mass, mass error, and fragment ions, is summarized for SHZ and SMJ in the Supplementary File S2. Representative components from three key classifications—phenylpropanoids, flavonoids, and iridoids were selected to elucidate the mass spectrometry fragmentation patterns (Figure 5).

#### 2.2.1. Identification of the Phenylpropanoids

Phenylpropanoids are predominantly sourced from SHZ, with quinic acid derivatives representing the most prevalent components. The quinic acid derivatives are predominantly characterized by quinic acid esters bearing aromatic substituents, specifically acylated derivatives of trans-cinnamic acids—primarily caffeoyl, feruloyl, sinapic acid, and p-coumaroyl moieties [23]. Dicaffeoylquinic acids, which are esterified derivatives of quinic acid substituted with two caffeoyl moieties, comprise a series of isomers, including 1,3-dicaffeoylquinic acid, 3,4-dicaffeoylquinic acid, 4,5-dicaffeoylquinic acid, 3,5-dicaffeoylquinic acid, 1,4-dicaffeoylquinic acid, and 1,5-dicaffeoylquinic acid, all of which exhibit identical fragmentation patterns. In the negative ion mode of SHZ, the deprotonated molecular ion [M-H]^−^ was observed for peaks SHZ_17_, SHZ_25_, SHZ_27_, SHZ_28_, SHZ_30_, and SHZ_31_ at *m*/*z* 515.1196 (C_25_H_24_O_12_). The fragmentation pattern *m*/*z* 353.0879 [M-H-C_9_H_6_O_3_]^−^ and *m*/*z* 335.0774 [M-H-C_9_H_6_O_3_-H_2_O]^−^, were consistent with the loss of the caffeoyl moiety and subsequent water molecule. Taking 3,4-dicaffeoylquinic acid as an example, its possible MS fragmentation pathway was illustrated in Figure 5A.

#### 2.2.2. Identification of the Flavonoids

Taking the peak labeled as both SHZ_40_ and SMJ_45_ as an example, it displayed an [M-H]^−^ ion at *m*/*z* 285.0407 (C_15_H_10_O_6_). The resulting fragments include 257.0452 [M-H-CO]^−^, 241.0508 [M-H-CO_2_]^−^, 199.0400 [M-H-C_2_H_2_O-CO_2_]^−^, and RDA fragment ions 133.0294, 151.0037. The compound was identified as luteolin based on comparison with an authentic reference standard and its MS/MS fragmentation data, which were consistent with those described in the literature. The *m*/*z* 151 (A ^1,3−^) ion can be used as a characteristic diagnostic ion for components containing 5,7-dihydroxy groups in the A ring of flavonoids, further verifying the identification. The possible cleavage pathway of luteolin is shown in Figure 5B.

#### 2.2.3. Identification of the Iridoids

Peaks SMJ _9, 10, 11, 17, 20, 22_ are a series of isomers with a molecular weight of 516.1485, producing an [M-H]^−^ parent ion at *m*/*z* 515.1406 in negative ion mode. Xcalibur software 4.1 suggests that its molecular formula is C_22_H_28_O_14_.The characteristic fragment ions 335.0776 [M−H-glc]^−^, 153.0193 [C_7_H_6_O_4_-H]^−^ were consistent with those of verproside (peak No. 24 in SMJ), whose cleavage scheme is shown in Figure 5C. The results confirmed that the compound was an iridoid structurally analogous to verproside. Based on the above data and comparison with the PubChem database, we propose that this compound is a cyclopentane-type iridoid, namely [6,7-dihydroxy-7-(hydroxymethyl)-1-[3,4,5-trihydroxy-6-(hydroxymethyl)oxan-2-yl]oxy-4a,5,6,7a-tetrahydro-1H-cyclopenta[c]pyran-5-yl] 3,4-dihydroxybenzoate and its isomers, for which limited literature is currently available and which were not found in the database of SMJ and its genus. In this study, we abbreviated it as compound-**1**.

Based on the mass fragmentation pattern of shanzhiside (peak No.3 in SMJ), a representative cyclopentane-type iridoid, it can be concluded that during the fragmentation process, the carbonyl group on the parent nucleus readily undergoes an intramolecular cyclization reaction with the hydroxyl group, generating a characteristic fragment with a *m*/*z* 211.0614 (Figure 5D). The fragment ion at *m*/*z* 225.0408 [M-H-Glc-C_6_H_8_O_3_]^−^ in Compound-1 is exactly the product of this cyclization reaction, further verifying the structural plausibility of Compound-**1**. The corresponding possible MS fragmentation pathway for this process is illustrated in Figure 5E.

### 2.3. Identification of XZZTP Transdermal Components

After 24 h of percutaneous penetration in an vitro Franz diffusion experiment, 52 transdermal components were identified in the transdermal sample based on retention time and mass spectrometry fragmentation pattern comparisons with the SHZ and SMJ mass spectrometry data.

**Figure 6:**
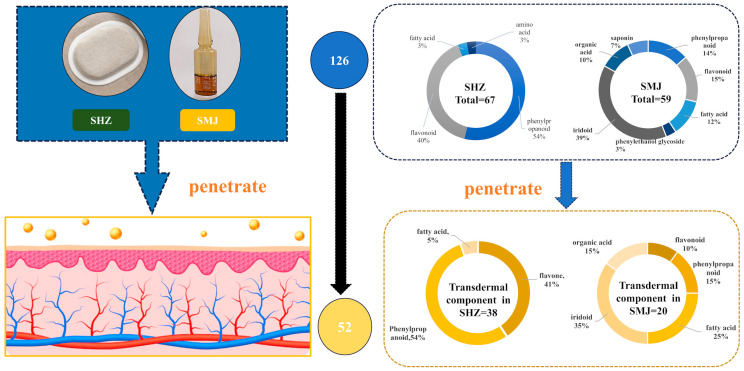
Number and type of XZZTP and transdermal sample components.

The TIC of XZZTP transdermal sample is shown in Figure S1. The identified transdermal components mainly comprised 21 phenylpropanoids, 15 flavonoids, seven iridoids, six fatty acids, three organic acids, and an amino acid (Figure 6), with the corresponding details documented in the Supplementary File S2.

These components may be the potential active components of XZZTP in bacterial inflammation by topical application and are intended for network pharmacology analysis in subsequent research.

### 2.4. Network Pharmacology Analysis Results

#### 2.4.1. Analysis of Components and Disease Targets

**Figure 7:**
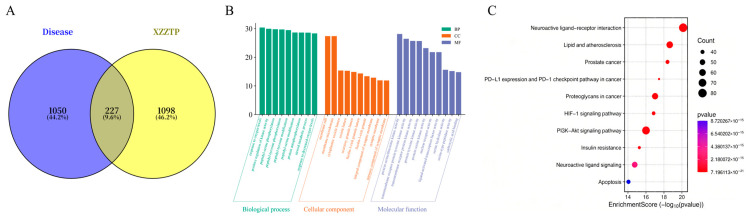
Network pharmacology analysis predicting the potential mechanisms for XZZTP treatment of bacterial inflammation. (**A**) Venn diagram. (**B**) GO enrichment. (**C**) KEGG enrichment.

To systematically elucidate the mechanism for XZZTP against bacterial inflammation, this study employed network pharmacology as a preliminary investigative approach. Initially, 1277 potential disease-related targets associated with “bacterial inflammation” were identified (File S3, Table S1). These were combined with the 1326 targets of the transdermal components of XZZTP (File S3, Table S2), yielding 227 overlapping targets. A Venn diagram was generated to visualize the intersection of these target sets (Figure 7A).

#### 2.4.2. GO and KEGG Enrichment Results

We performed GO and KEGG analyses of the overlapping targets [24]. It showed that the numbers of CC, MF, and BP were 519, 945, and 5636, respectively (File S3, Table S3). According to the lowest q-value, the top 10 significantly enriched terms of BP, CC, and MF are listed in Figure 7B. In BP, the targets were mainly involved in positive regulation of kinase activity, peptidyl–serine phosphorylation, peptidyl–tyrosine phosphorylation, peptidyl–tyrosine modification, peptidyl–serine modification, protein autophosphorylation, steroid metabolic process, response to oxygen levels, response to decreased oxygen levels, and protein serine/threonine kinase activity. In CC, the targets were mainly involved in membrane rafts, membrane microdomains, cytoplasmic vesicle lumen, vesicle lumen, secretory granule lumen, ficolin-1-rich granule lumen, ficolin-1-rich granules, integral component of the synaptic membrane, the synaptic membrane, and intrinsic components of the synaptic membrane. In MF, the targets were mainly involved in protein serine/threonine kinase activity, transmembrane receptor protein tyrosine kinase activity, transmembrane receptor protein kinase activity, protein tyrosine kinase activity, protein serine kinase activity, nuclear receptor activity, ligand-activated transcription factor activity, serine hydrolase activity, serine-type peptidase activity, and carboxylic acid binding.

Furthermore, the KEGG pathway enrichment analysis yielded 332 enriched pathways (File S3, Table S4). The top 10 pathways, ranked by the q-value, are presented in a bubble chart. (Figure 7C). Notably, both the PI3K-AKT signaling pathway and the HIF-1 signaling pathway were significantly enriched. These signaling pathways are closely associated with the diseases related to bacterial inflammation indicated for XZZZTP, such as diarrhea [25], lung bacterial inflammation [26,27] bronchitis [26], and pneumonia [28,29]. In bacterial inflammation, the PI3K-AKT pathway promotes the release of pro-inflammatory factors and inhibits the apoptosis of immune cells to prolong the inflammatory response [27]. Meanwhile, the sustained inflammatory response leads to tissue hypoxia, which in turn activates the HIF-1 signaling pathway, thereby contributing to the pathogenesis of the disease. The PI3K/AKT and HIF-1 pathways crosstalk via hypoxia and metabolism to form the synergistic pro-inflammatory PI3K/AKT/HIF-1 axis [30].

Based on the above evidence, we propose that XZZTP may exert therapeutic effects on bacterial inflammation by modulating the PI3K/AKT/HIF-1 signaling pathway to inhibit excessive bacterial inflammation and enhance immune regulation, providing a clear theoretical direction for further experimental validation and mechanistic elucidation.

### 2.5. In Vitro Experiment Validation

Although LPS does not recapitulate the full complexity of live bacterial infection, its ability to activate the TLR4-mediated PI3K/AKT/HIF-1 signaling axis makes it an ideal model for investigating the mechanism by which the drug suppresses infection-associated inflammation. Therefore, we selected core proteins within this pathway for expression analysis in this model following XZZTP intervention, aiming to validate the signaling pathway predicted by network pharmacology. The experimental conditions were the same as those in the Section 2.1.2. The p-PI3K, p-AKT, and HIF-1α were selected for Western blotting validation.

**Figure 8:**
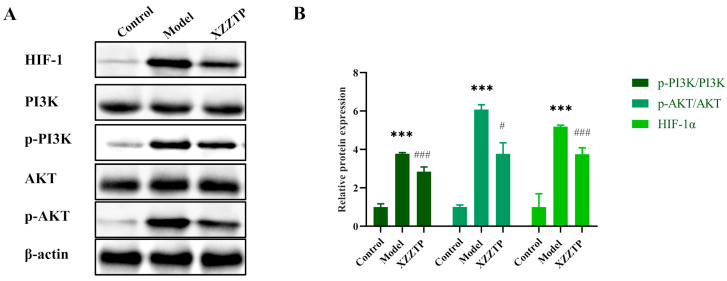
Experimental validation results. Western blotting analysis of the protein expression levels of PI3K, phosphorylated PI3K (p-PI3K), AKT, phosphorylated AKT (p-AKT), HIF-1α, and β-actin in the RAW 264.7 macrophages. (**A**). Quantification of p-PI3K/PI3K, p-AKT/AKT, and HIF-1α based on the Western blotting images was performed via Image J 1.54p in the RAW 264.7 macrophage (**B**). Data are presented as the mean ± SD (*n* = 3). *** *p* < 0.001 vs. the control group; # *p* < 0.05, ### *p* < 0.001 vs. the model group.

As shown in Figure 8, compared with the control group, the expression of p-PI3K, p-AKT, and HIF-1α in the model group was significantly increased (all *p* < 0.001). Compared with the model group, the expression of p-PI3K/PI3K (*p* < 0.001), p-AKT/AKT (*p* < 0.05), and HIF-1α (*p* < 0.001) showed a significant decrease in the XZZTP group. This indicated that XZZTP can attenuate excessive inflammatory expression by downregulating the PI3K/AKT/HIF-1 pathway.

### 2.6. Molecular Docking Screening

**Figure 9:**
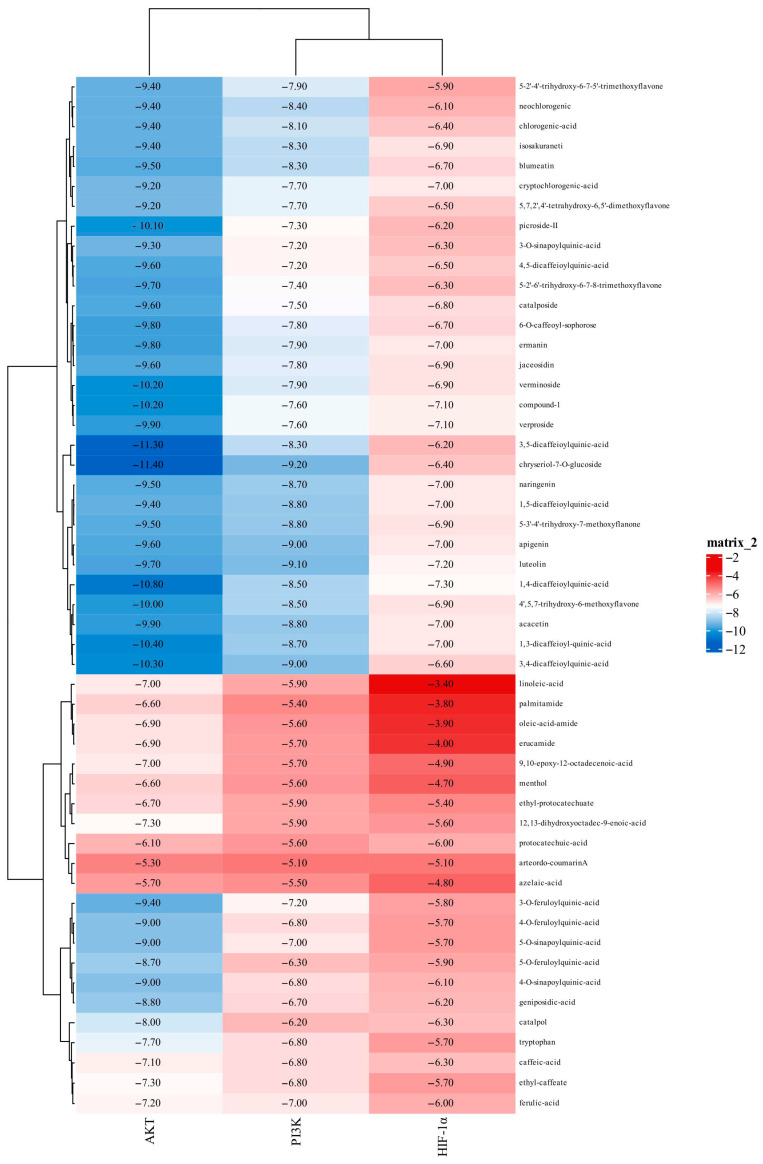
Molecular docking scores of the 52 components with PI3K, AKT, and HIF-1 α.

**Figure 10:**
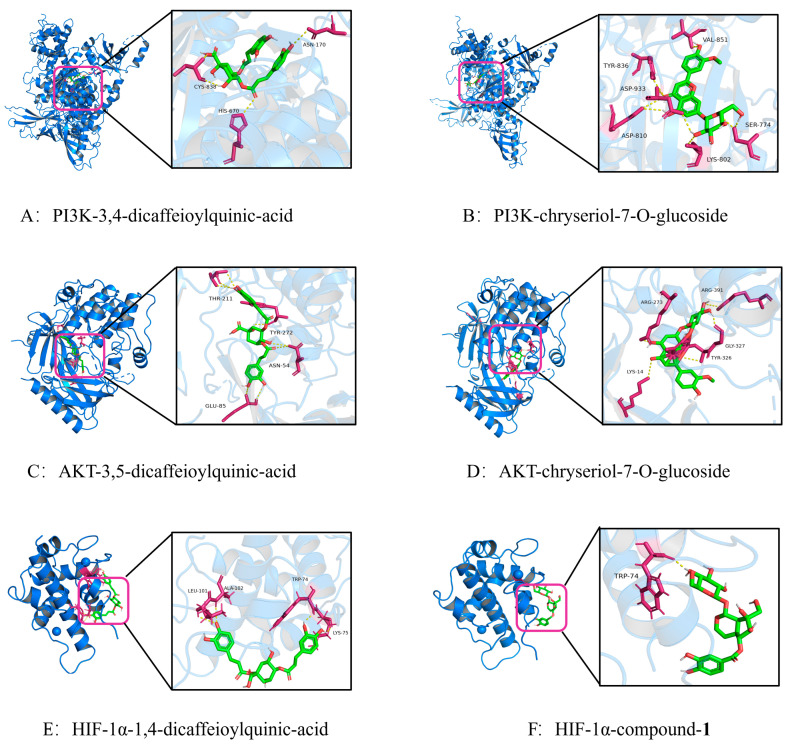
Three-dimensional molecular docking analysis of active compounds with target proteins. (Green sticks: ligand molecules; magenta sticks: interacting amino acid residues; yellow dashed lines: hydrogen bonds).

To identify the active components underlying the anti-bacterial-inflammation effects of XZZTP, molecular docking was performed to screen 52 transdermal components against PI3KCA, AKT1, and HIF-1α/CBP p300 (core proteins of the PI3K/AKT/HIF-1α signaling pathway), with binding energy serving as the screening criterion. Drugs against these targets that are clinically approved or in clinical trials were used as positive controls [31]. Components with binding energies lower than the average value of the corresponding positive controls were identified as primary active components mediating the therapeutic effect of XZZTP against bacterial inflammation. All RMSD < 2.0 Å proves that the docking results were accurate and reliable. The results of molecular docking are shown in Figure 9. The binding energy information of proteins with positive controls is shown in Table 2. To visualize the interactions, active components from each classification was selected for molecular docking with PI3K, AKT, and HIF-1α, and the results are presented in Figure 10. The number and the corresponding binding amino acid residues of hydrogen bonds formed between these proteins and the active components in XZZTP are presented in Table 3.

Molecular docking analysis revealed that 3,5-dicaffeoylquinic acid, 3,4-dicaffeoylquinic acid, 1,4-dicaffeoylquinic acid, chrysoeriol-7-O-glucoside, apigenin, luteolin, compound-1, and verproside all showed binding energies that were lower than those of the positive control against their corresponding targets, suggesting that they were the active components responsible for the pharmacological effects of XZZTP in practical applications. Specifically, 3,4-dicaffeoylquinic acid exhibited a binding energy of −9.0 kcal/mol with PI3K; 3,5-dicaffeoylquinic acid bound to AKT with binding energies of −11.3 kcal/mol; 1,4-dicaffeoylquinic acid interacted with HIF-1α with a binding energy of −7.3 kcal/mol in the Table 3. These findings suggest that dicaffeoylquinic acid may serve as the major active components responsible for the anti-bacterial inflammation effects of XZZTP. In addition, the newly identified component in XZZTP-Compound-**1** also exhibited low binding energy for the core target in molecular docking assays, with a strong binding energy of −7.1 kcal/mol to HIF-1α. These findings suggest that Compound-**1** and its isomers are the active components responsible for mediating the anti-bacterial inflammation activity of XZZTP.

### 2.7. MD Simulation Validation

To further validate the binding stability of active components with core proteins under physiological dynamic conditions, three low binding energy receptor–ligand (with different component classes)complexes were selected from the molecular docking results, including PI3K-Chrysoeriol-7-O-glucoside, AKT-3,5-dicaffeoylquinic acid, and HIF-1α-compound-**1**, and subjected to 100 ns molecular dynamicssimulations initiated from the corresponding docking poses. Notably, these ligands were derived from different types of components. Root Mean Square Deviation (RMSD), Root Mean Square Fluctuation (RMSF), and Radius of Gyration (Rg) were employed as core parameters to evaluate the dynamic behavior of the complexes. RMSD was calculated to assess the global conformational stability and simulation convergence, RMSF was used to analyze the local flexibility of individual residues, and Rg was applied to monitor the structural compactness and folding state of the proteins. In addition, the MM-PBSA binding free energy was calculated to quantitatively evaluate the binding affinity between ligands and the target proteins. This multi-parameter analysis comprehensively characterized the effects of ligand binding on protein conformational dynamics, verifying the plausibility and stability of the complex interactions.

**Figure 11:**
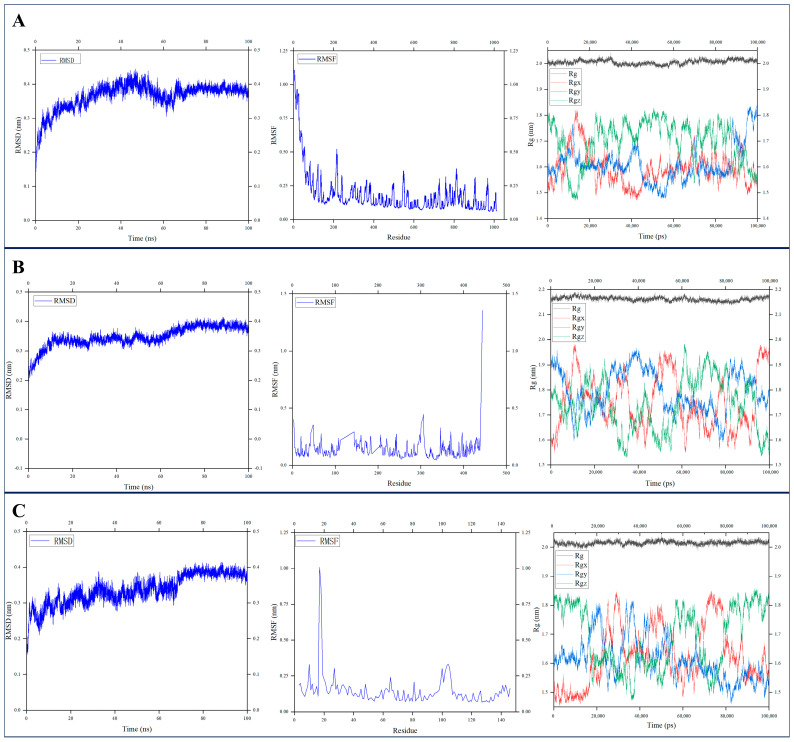
RMSD, RMSF and Rg analyses of three core protein–component complexes during 100 ns molecular dynamics simulation. (**A**) PI3K-Chrysoeriol-7-O-glucoside complex; (**B**) AKT-3,5-dicaffeoylquinic acid complex; (**C**) HIF-1α-Compound-**1** complex.

For the PI3K-Chrysoeriol-7-O-glucoside complex, RMSD fluctuated between 0.3 and 0.45 nm, with an overall amplitude < 0.15 nm. The residues in the core functional region fluctuated mostly below 0.5 nm (except for the more flexible N-terminal), with no obvious flexibility at the core binding interface. The average Rg remained stable at 2.0–2.05 nm. The MM-PBSA binding energy of this complex was −29.58 kcal/mol (Figure 11A).

For the AKT-3,5-dicaffeoylquinic acid complex, RMSD fluctuated between 0.2 and 0.4 nm (with an overall amplitude < 0.2 nm). Most residues fluctuated below 0.5 nm (only a prominent peak at the C-terminal tail), and the core binding interface showed no marked flexibility; the average Rg was 2.15–2.20 nm. The MM-PBSA binding energy of this complex was −32.52 kcal/mol (Figure 11B).

For the HIF-1α-Compound-1 complex, RMSD fluctuated between 0.3 and0.4 nm, with an overall amplitude <0.1 nm. The core domain residues fluctuated below 0.5 nm (only a notable flexible peak at the N-terminal), and the core binding interface remained rigid; the average Rg was stably maintained at 2.0–2.05 nm. The MM-PBSA binding energy of this complex was −11.15 kcal/mol (Figure 11C).

In summary, the results of the 100-ns molecular dynamics simulations verified the binding stability between the active components screened by molecular docking technology and their corresponding proteins, further supporting the reliability of these components as core active components.

## 3. Discussion

XZZTP has been used clinically for over 20 years. Owing to its therapeutic effects, as well as its convenience and safety, it has gained widespread recognition among patients. There are numerous clinical reports on its combination with other drugs. However, there is currently little literature on the specific pharmacological effects of XZZTP, and its pharmacological mechanisms and active components have yet to be systematically and scientifically elucidated. Our findings demonstrate that XZZTP can alleviate inflammation both in vivo and in vitro. Through an integrated strategy combining LC-MS/MS, in vitro Franz diffusion assays, and network pharmacology, we characterized the constituent components and transdermal components of XZZTP and elucidated that the PI3K/AKT/HIF-1α pathway serves as its core anti-inflammatory signaling pathway. Furthermore, 3,5-dicaffeoylquinic acid, 3,4-dicaffeoylquinic acid, 1,4-dicaffeoylquinic acid, chrysoeriol-7-O-glucoside, apigenin, luteolin, Compound-**1,** and verproside were identified as active components of XZZTP by molecular docking.

This study first aimed to scientifically elucidate the anti-inflammatory effects of XZZTP using two models: xylene-induced ear swelling in vivo and LPS-induced bacterial inflammation in RAW 264.7 macrophages in vitro. The positive control XTTP has been documented to exert well-defined anti-inflammatory pharmacological effects, demonstrating significant efficacy in alleviating inflammation [32]. Literature reports indicate that its potency in modulating the inflammatory mediator NO was comparable to the results obtained in the present study, thereby supporting the reliability of our experimental findings [33]. In terms of degree of ear swelling and the inhibition rates of inflammatory cytokines, XZZTP showed a significant anti-inflammatory activity. There was no statistically significant difference in the anti-inflammatory efficacy between XZZTP and the positive control XTTP.

There have been no previous reports on the analysis of the components in XZZTP using LC-MS/MS methods. A total of 126 components were identified by LC-MS/MS, including 59 previously unreported components in SHZ and 37 in SMJ. This work significantly enriches their component databases. SHZ was found to contain a considerable number of quinic acid derivatives and flavonoid isomers. These compounds represent the major components of SHZ but also pose significant challenges for their chemical identification. Meanwhile, SMJ is predominantly composed of iridoids. Notably, Compound-**1** and its series of isomers, newly identified in SMJ, have not been reported before. Further extraction and isolation are required for confirmatory validation.

The main active components identified in XZZTP include phenylpropanoids, iridoids, and flavonoids. To date, no obvious toxicity of these components has been reported in the literature. Raw 264.7 macrophage viability experiments also confirmed that XZZTP exhibited no significant cytotoxicity. Furthermore, no obvious adverse reactions or toxic side effects were observed in clinical observations of its combined application. Taken together, XZZTP exhibits a favorable safety profiles for long-term use.

To elucidate the pharmacodynamic mechanism of XZZTP, this study identified 52 potential active components by LC-MS/MS in an in vitro transdermal sample. Based on these findings, network pharmacology further revealed that these components exert holistic therapeutic effects through synergistic interactions. The main transdermal components of SHZ are phenylpropanoids and flavonoids, while the main transdermal components of SMJ, including iridoids, flavonoids, phenylpropanoids, organic acids, and fatty acids, can be absorbed through the skin as potential active components. Compound-**1** can permeate the skin, indicating its potential as an active component of XZZTP. Its functional role merits further elucidation.

The KEGG pathway enrichment analysis revealed significant enrichment of both the PI3K-AKT and HIF-1 signaling pathways, highlighting their central role in the pharmacological mechanism of XZZTP against bacterial inflammation. The PI3K-AKT pathway acts as a key signaling hub in the inflammatory response. Upon detection of inflammatory stimuli, this pathway is activated, then promotes bacterial inflammation through a dual mechanism: directly, by phosphorylating transcription factors to amplify inflammatory signaling [27]; and indirectly, by enhancing the synthesis and activity of hypoxia-inducible factor HIF-1α via mTOR activation [30]. This establishes a critical link between inflammatory signaling and metabolic reprogramming. Conversely, hypoxia associated with bacterial inflammation directly stabilizes HIF-1α. Furthermore, the accumulation of HIF-1α triggers pro-inflammatory metabolic reprogramming, characterized by elevated glycolysis, which supplies sufficient energy and biosynthetic precursors to sustain the functional activity of inflammatory macrophages and collaboratively amplifies inflammatory signaling with PI3K-AKT [28]. Thus, the PI3K-AKT/HIF-1α pathway promotes metabolic reprogramming and sustains persistent bacterial inflammation, serving as a key link between inflammatory signaling, metabolic adaptation, and disease progression.

Phosphatidylinositol 3-kinase catalytic subunit alpha (PI3KCA), as an upstream key kinase of the PI3K-AKT pathway, catalyzes the generation of the second messenger PIP3, providing the essential molecular platform for membrane recruitment and activation of downstream effector proteins, serving as the central engine that initiates pathway signal transduction [34]. Protein kinase B (AKT), as the core central kinase of the PI3K-AKT pathway, converts upstream PIP3 lipid signaling into broad cellular biological effects. Through phosphorylation, it regulates downstream key targets and directly drives cell survival, proliferation, growth, and metabolism [35]. Hypoxia-inducible factor 1-alpha (HIF-1α) is the core regulatory subunit of the HIF-1 pathway, whose stability is directly governed by oxygen concentration. As the central transcriptional hub for cellular hypoxia sensing and response, HIF-1α undergoes stabilization and dimerization, thereby driving the expression of a broad spectrum of downstream target genes and orchestrating adaptive survival and functional reprogramming of cells under hypoxic conditions [36]. Western blotting experiments showed that XZZTP could significantly reduce the expression of p-PI3K, p-AKT, and HIF-1 α in the RAW 264.7 macrophages, indicating that XZZTP relies on the PI3K/AKT/HIF-1α key signaling pathway to coordinate downstream inflammatory gene expression and glycolysis reprogramming.

Molecular docking screening directly predicts the binding affinity between components and targets through three-dimensional structural simulation. This approach does not rely on prior knowledge and holds significant potential for new drug discovery. A total of eight components with lower binding energies than the positive control were identified, which represent the key anti-inflammatory active components of XZZTP, acting via the PI3K/AKT/HIF-1α signaling pathway.

Given that diarrhea represents the primary clinical indication of XZZTP, its therapeutic efficacy is likely attributed to the systemic absorption of active ingredients that modulate the intestinal inflammatory microenvironment. In the present study, eight core components were identified, all of which have been previously documented to exert anti-inflammatory activities. Notably, luteolin [37,38] and 3,5-dicaffeoylquinic acid [39] have been reported to regulate the PI3K/AKT/HIF-1α signaling pathway. Considering the pivotal role of this signaling axis in the pathogenesis of diarrhea, the presence of these two components further supports the therapeutic relevance of XZZTP in managing this condition. Additionally, apigenin [40], 1,4-dicaffeoylquinic acid [41], and verproside [42] have been well characterized for their potent anti-inflammatory effects in previous studies. The potential anti-inflammatory properties of chrysoeriol-7-O-glucoside and Compound-**1** are supported by their structural analogs, chrysoeriol [43] and shanzhiside [44], which have been experimentally verified to inhibit inflammatory responses and mitigate oxidative stress. Thus, the collective anti-inflammatory pharmacological activities exhibited by these active components provide a solid mechanistic foundation for the enhanced therapeutic efficacy of the other powder when administered in combination.

This study preliminarily confirms that XZZTP exhibits significant anti-inflammatory activities. XZZTP may exert therapeutic effects by modulating the PI3K-AKT-HIF-1 pathway, and the primary active components include three kinds of dicaffeoylquinic acid derivatives, chrysoeriol-7-O-glucoside, apigenin, luteolin, compound-**1,** and verproside, which suppress bacterial inflammation. In summary, this study comprehensively elucidated the pharmacological mechanism and primary active components of XZZTP against bacterial inflammation, providing an experimental basis for its clinical application and enriching the theoretical understanding of its pharmacological properties.

## 4. Materials and Methods

### 4.1. Instruments

The instruments included an Xcalibur 4.1 mass spectrometry workstation, a Vanquish ultrahigh-performance liquid chromatography system equipped with a quadruple gradient pump, an autosampler, a column warming chamber, a DAD detector (Thermo Fisher Scientific, Waltham, MA, USA), a CO_2_ incubator (In-VitroCell NU-5731, Nuaire. Co., Ltd., London, UK), a NU-543-600S Biosafety cabinet (Nuaire. Co., Ltd., London, UK), a TK-12D transdermal system (Shanghai Yuyan Scientific Instrument Co., Ltd., Shanghai, China), a KQ-500DE numerical control ultrasonic cleaner (Kunshan Ultrasonic Instrument Co., Ltd., Suzhou, China), an SQP electronic balance (Beijing saidoris Scientific Instrument Co., Ltd., Beijing, China), an IX73 inverted fluorescence microscope (Olympus Corporation, Tokyo, Japan), Multiskan microplate reader (BioTek Instruments, Inc., Winooski, VT, USA), and a BLT 6000Plus instrument (Biolight Biotechnology Co., Ltd., Guangzhou, China).

### 4.2. Reagents and Materials

(Batch number: 20240408) XZZTP (Yabao Pharmaceutical Group Co., Ltd., Yuncheng, China); Xiao Tong Tie Gao (Tong ren tang Pharmaceutical Co., Ltd., Beijing, China); LC-MS/MS methanol, acetonitrile (Thermo Fisher Scientific (China) Co., Ltd., Shanghai, China), Wahaha pure water (Wahaha Group, Hangzhou, China), and formic acid (Thermo Fisher Scientific (China) Co., Ltd., Shanghai, China) were used for the mobile phase, and the rest of the reagents were analytically pure; all TCM reference standard sources (File S3, Table S5); xylene (Macklin Biochemical Technology Co., Ltd., Dalian, China); CL-0190 RAW264.7 macrophages (Pricella Biotechnology Co., Ltd., Wuhan, China); Gibco DMEM and 1% penicillin/streptomycin (Thermo Fisher Scientific (China) Co., Ltd., Shanghai, China); fetal bovine serum (Corning Incorporated, Corning, NY, USA); LPS (Sigma-Aldrich (Shanghai) Trading Co., Ltd., Shanghai, China); TNF-α, PGE2 ELISA kits (Elabscience Technology Co., Ltd., Wuhan, China); CCK8 kits and Nitric Oxide Assay kits (Beyotime Biotech Inc., Shanghai, China); polyvinylidene fluoride (PVDF) membranes (Pall Corporation, Port Washington, NY, USA); antibodies against p-PI3K (#4228T), PI3K (#4249T), AKT (#4691T), p-AKT (#9271T), and HIF-1α (#14179T) (Cell Signaling Technology, Beverly, MA, USA); (Cat#66009-1-Ig) anti-β-actin (Proteintech Group, Inc., Wuhan, China).

### 4.3. Anti-Inflammatory Effect of XZZTP In Vivo and In Vitro

#### 4.3.1. Xylene-Induced Mouse Ear Edema

##### Animal Experiment

KM male mice weighing 18–22 g were bred in an environment of 22 ± 2 °C, 60 ± 10% relative humidity, and 12 h light/dark cycles, with free access to water and food. The experiments were performed after the mice adapted to the experimental environment for 7 days. The animal experimental license numbers are SYXK (Jing) 2023-0011.

The studies were carried out according to the previous reports [45]. A total of 30 km male mice were randomly allocated into three groups (*n* = 10 per group). An amount of 0.04 mL of xylene was evenly administered to the patched area of the right ear in every mouse, with the left ear serving as the untreated control. In both the XZZTP treatment group and the positive control group (treated with XTTP), a medicated patch of the same size (0.6 cm × 0.6 cm) was then applied to the right ear of the mouse. Each group of mice received topical administration for 1 h. Subsequently, all mice were euthanized after the patch application. Uniform circular ear samples (6 mm in radius) were then collected from the corresponding areas using a tissue punch, and each sample was weighed on an electronic balance. The degree of ear swelling (Δm) and the percentage swelling inhibition are given by Equations (1) and (2), respectively.

Δm = right ear mass − left ear mass,(1)

% Swelling inhibition = 100 × (1 − A_2_/A_1_) (2)

A_1_ = Degree of ear swelling of model group, A_2_ = Degree of ear swelling of other group.

##### H&E Staining Examination

Fresh mouse ear samples were fixed with 4% paraformaldehyde for 24 h, dehydrated, embedded in paraffin, sectioned, and stained. The degree of inflammatory cell infiltration and edema in the ear during the H&E staining examination was observed under a microscope and photographed for subsequent analysis (magnification, ×40).

#### 4.3.2. Cell Culture and LPS-Induced Bacterial Inflammation

##### Cell Culture

RAW 264.7 macrophages were cultured in Dulbecco’s Modified Eagle Medium (DMEM) supplemented with 10% (*v*/*v*) fetal bovine serum (FBS), 100 µg/mL streptomycin, and 100 U/mL penicillin and maintained at 37 °C in a humidified atmosphere of 5% CO_2_.

##### Cell Viability Assay

RAW 264.7 macrophages were seeded (5 × 10^5^ cells/well) and cultured in 96-well plates overnight. The supernatant was removed, and the cells were treated with various concentrations of XZZTP and XTTP (50, 100, 200, 400, 800, and 1000 µg/mL) for 24 h. The control group was treated with drug-free 10% FBS DMEM. After treatment for 24 h, the supernatant was removed, the CCK-8 solution (10 µL) was added to each well and incubated for 1 h at 37 °C. Finally, absorbance was measured at 450 nm using a microplate reader, and cell viability was calculated relative to the control group.

##### Determination of NO, TNF-α, PGE2 Concentration

The anti-inflammatory activity was evaluated in LPS-stimulated RAW 264.7 macrophages. Following a 2 h pretreatment with XZZTP (25, 50, 100, and 200 μg/mL), cells were co-incubated with LPS (1 μg/mL) for 8 h. The experimental groups were defined as follows: the model group (LPS only), the control group (10% DMEM), the XZZTP group, and the positive control group (XTTP). Each treatment was performed in triplicate. Following incubation, the culture supernatants were collected to quantitatively assess the levels of key inflammatory mediators—NO, TNF-α, and PGE2.

### 4.4. LC-MS/MS Analysis

#### 4.4.1. Instrument Conditions

LC-MS/MS analysis was conducted on the Q-Exactive-Orbitrap mass spectrometer coupled with a heated electrospray ionization (HESI) source. Chromatographic separation was performed on the Waters ACQUITY UPLC HSS T3 column (2.1 mm × 100 mm, 1.8 μm) maintained at 30 °C. The mobile phases consisted of 0.1% (*v*/*v*) formic acid in water (solvent A) and acetonitrile (solvent B). The flow rate was set at 0.3 mL/min, and the injection volume was 10 μL. The gradient process is as follows: 0–3 min, 5% B; 3–13 min, 5–30% B; 13–18 min, 30–40% B; 18–24 min, 40–60% B; 24–38, 60–100% B; 38–44 min, 100% B.

Positive ion mode: capillary voltage 3500 V, capillary temperature 400°C, ion source temperature 320 °C, mass spectrometry acquisition range *m*/*z* 100~1500, resolution 70,000, and S-Lens RF Level 55. Negative ion mode, capillary voltage 3000 V, capillary temperature 400 °C, ion source temperature 320 °C, mass spectrometry acquisition range *m*/*z* 100~1500, resolution 70,000, and S-Lens RF Level 55. Step collision energy 20, 40, and 60 V.

#### 4.4.2. Mass Spectrometry Identification Strategy

In order to more comprehensively analyze the chemical components of the XZZTP, the SHZ and SMJ samples were extracted and analyzed by LC-MS/MS, respectively. To characterize the chemical components of SHZ and SMJ, which have rarely been studied previously, we employed LC-MS/MS to analyze their components and transdermal components. The molecular weight was determined with high precision based on quasi-molecular ions such as [M-H]^−^ and [M + H]^+^, achieving a mass accuracy of 10 ppm. Structural identification was carried out by fragment ions and comparison of their retention times with those of reference standards.

### 4.5. Samples Preparation

#### 4.5.1. XZZTP Samples Preparation

One piece of SHZ medicinal patch was cut into small pieces and ultrasonically extracted with 50% ethanol for 30 min at room temperature. The supernatant was filtered through a 0.22 µm micron filter membrane for LC-MS/MS analysis. The SMJ liquid in the ampoule bottle was shaken well and then filtered through a 0.22 µm micron filter membrane for LC-MS/MS analysis.

#### 4.5.2. XZZTP Transdermal Samples Preparation

In vitro Franz diffusion experiments are advantageous due to their rapid execution and procedural simplicity. A TK-12D transdermal system, equipped with vertical diffusion cells with an effective penetration area of 3.14 cm^2^ was adopted. The same appropriately sized XZZTP was placed on the stratum corneum of clean abdominal skin excised from male Sprague–Dawley rats in the supply pool. Phosphate-buffered saline (PBS) was conventionally employed as the receptor solution in this study. Approximately 8 mL of PBS was added to the receiving pool and mixed at a speed of 300 r/min, with the temperature kept at (37 ± 0.2) °C. After 24 h, the receiving solution was collected. Skin samples were collected, rinsed with physiological saline, and homogenized in 1 mL of methanol, followed by centrifugation (12,000 rpm, 10 min) to collect the supernatant. The components that permeated through the skin into the receptor fluid and those retained within the skin were collectively investigated as the transdermal components. The samples were prepared for LC-MS/MS analysis by filtration through 0.22 µm filter membranes.

### 4.6. Network Pharmacology Analysis

#### 4.6.1. Protein Targets of Components

The 52 transdermal components were taken as potential active components in XZZTP, and their protein targets in Homo sapiens were predicted by using TCMSP (https://tcmsp-e.com/tcmsp.php, accessed on 10 August 2024), Pharm Mapper (https://stitch.embl.de, accessed on 11 August 2024), and Swiss Target Prediction (https://www.swisstargetprediction.ch/, accessed on 11 August 2024). The targets derived from these databases were merged and deduplicated to obtain the complete set of targets for the components. 

#### 4.6.2. Protein Targets of Disease

Using “bacterial inflammation” as keywords, the predicted targets were obtained from TTD (https://ttd.idrblab.cn/, accessed on 21 August 2024); DisGeNET (https://www.disgenet.org, accessed on 21 August 2024); GeneCards (https://www.genecards.org, accessed on 21 August 2024). Finally, take all targets from each database with values greater than the median and merge them to remove duplicates to obtain disease-related targets.

#### 4.6.3. GO and KEGG Enrichment 

The Venny 2.1 database (https://bioinfogp.cnb.csic.es/tools/venny/index.html, accessed on 21 August 2024) was utilized to identify overlapping genes between component targets and disease targets. The GO database offers a standardized classification system for gene annotation across three primary categories: cellular component (CC), molecular function (MF), and biological process (BP). Meanwhile, the KEGG knowledge base provides a systematic framework for understanding gene functions and their involvement in biological pathways. GO biological function analysis and KEGG enrichment analysis were performed by bioinformatics (https://bioinformatics.com.cn/, accessed on 20 December 2025), an integrated tool for functional annotation and interpretation of omics date to perform GO and KEGG enrichment analysis. The q-value threshold was set to 0.05 to verify that the channel calculation results were statistically significant.

### 4.7. Protein Expression Determination by Western Blotting

The cell culture conditions are described in Section 4.3.2. Following a 2 h pretreatment with XZZTP (200 μg/mL), cells were co-incubated with LPS (1 μg/mL) for 8 h. The cell supernatant was discarded, and the RAW 264.7 macrophage samples were lysed in cold lysis buffer (comprising 50 mM pH 7.4 Tris, 150 mM NaCl, 1% Triton X-100, 1% sodium deoxycholate, 0.1% SDS, 1% PMSF, and phosphatase inhibitors) for 30 min and centrifuged (12,000× *g*, 10 min, 4 °C). Then, the lysates were separated by SDS–PAGE and electrophoretically transferred onto polyvinylidene fluoride membranes. The membranes were blocked in 5% BSA for 2 h, then incubated with a primary antibody for PI3K (1:1000), p-PI3K (1:500), AKT (1:1000), p-AKT (1:1000), HIF-1α (1:1000), and β-actin (1:5000) at 4 °C overnight and subsequently with horseradish peroxidase (HRP)-conjugated secondary antibodies at room temperature for 1 h. Finally, immunoreactive protein bands were visualized using an Enhanced Chemiluminescence (ECL) kit and analyzed with a gel imager.

### 4.8. Active Components Identification via Molecular Docking

We used the PubChem database to search the three-dimensional structure of 52 transdermal components of XZZTP. The core proteins in the key pathway were selected as protein receptors for molecular docking. The 3D structures of the targets were obtained from the RCSB PDB database (https://www.rcsb.org/, accessed on 29 December 2025). For molecular docking, proteins were pretreated using AutoDock Tools 1.5.7, including water removal and hydrogen addition. The ligands were also prepared in AutoDock Tools, including the addition of hydrogen, Gasteiger charges, detecting root, and choosing torsions. Then, the GetBox Plugin in PyMOL 2.6 was used to predict the active pockets of the proteins. Finally, Auto Dock Vina 1.1.2 was used to implement docking simulation. This study searched the IUPHAR/BPS Guide to PHARMACOLOGY database (https://www.guidetopharmacology.org/) and the literature for target inhibitors. The average binding energy derived from the docking results was used as benchmark thresholds for active components. Components in XZZTP with binding energies lower than the average of the inhibitors were considered as active components of XZZTP.

PyMOL is an open source molecular modeling visualization software, which can analyze the binding situation of receptor proteins and ligands in molecular docking and mark hydrogen bonds. Complexes of the strongest binding components with the 3 targets were imported into PyMOL 2.6e for further visualization. The Python environment on which the above software depends is Python (3.7.4).

### 4.9. MD Simulation

MD simulations were conducted to verify ligand–receptor complex binding stability and optimize molecular docking predictions. Specifically, 100 ns MD simulations were performed on low binding energy complexes (screened via molecular docking) using Gromacs 2020.4: the CHARMM force field was used for proteins, and Amber force field parameters for small molecules were generated via the ACPYPE server to construct a complete topology file. Complexes and isolated proteins were embedded in 12 Å cubic periodic boxes, solvated with the TIP3P water model, and neutralized with Na^+^/Cl^−^ counterions. After a 50,000-step steepest descent energy minimization, equilibration was performed (100 ps NVT heating to 300 K, 100 ps NPT at 1 atm), followed by 100 ns simulations (300 K, 1 atm, 2 fs time step), with coordinates saved every 10 ps.

Post-simulation, trajectory files were processed, and complex stability, residue flexibility, and conformational compactness were evaluated using RMSD, RMSF, Rg, Rgx, Rgy, and Rgz. Binding free energy (ΔGbind) was calculated via the GROMACS-integrated g_mmpbsa tool (MM-PBSA method) to quantify intermolecular interaction strength.

### 4.10. Statistics

Data were preliminarily organized and calculated using Excel. Statistical analysis was performed using ANOVA analysis with SPSS 20.0. When variances were homogeneous (*p* > 0.05), LSD post-hoc tests were selected; when variances were heterogeneous (*p* < 0.05), Dunnett’s T3 post-hoc tests were chosen. GraphPad Prism 8.0 software was used for data visualization, with results expressed as means ± SD.

## Figures and Tables

**Table 1:** Inhibitory effect of XZZTP on xylene-induced ear swelling in mice ($\bar{x}$ ± s, *n* = 10).

Groups	Degree of Ear Swelling (mg)	Swelling Inhibition (%)
Model group	4.230 ± 0.475	—
Positive control group	2.068 ± 0.431 **	49.93
XZZTP group	2.24 ± 0.539 **	45.96

Compared with the model group: ** *p* < 0.01.

**Table 2:** Binding energies of positive controls against the PI3K, AKT, and HIF-1 α.

Target	Positive Controls	Binding Energies/(kcal·mol^−1^)	Average/(kcal·mol^−1^)
PI3K	Izorlisib	−8.1	−8.87
	Copanlisib	−9.1	
	Taselisib	−9.4	
AKT	Capivasertib	−10.8	−11.1
	Lpatasertib	−10.3	
	MS170	−12.2	
HIF-1α	Calactin	−7.3	−7.07
	Glyceollin II	−7.2	
	NNC	−6.7	

**Table 3:** Molecular docking scores of the active components of XZZTP with PI3K, AKT, and HIF-1 α.

Protein	Grid Center and Grid Size	Component	Classification	Binding Energies/(kcal.mol^−1^)	No. H-Bond	Amino Acid Residue with H-Bond
PI3K (PDB ID: 4L23)	Center: (32.6, 45.3, 42.1) Size: (40, 40, 40)	3,4-dicaffeoylquinic-acid	phenylpropanoid	−9	3	HIS-670, ASN-170, CYS-838
		Chrysoeriol-7-O-glucoside	flavonoid	−9.2	5	VAL-851, TYR-836, ASP-810, ASP-933, SER-774
		Apigenin	flavonoid	−9.1	3	VAL-851, TYR-836, ASP-933
		Luteolin	flavonoid	−9	3	VAL-851, TYR-836, ASP-933
AKT (PDB ID: 6HHI)	Center: (−2.8, 6.2, −10.7) Size: (40, 40, 40)	3,5-dicaffeoylquinic-acid	phenylpropanoid	−11.3	4	THR-211, TYR-272, ASN-54, GLU-85
		Chrysoeriol-7-O-glucoside	flavonoid	−11.4	5	LYS-14, TYR-326, ARG-273, GLY-327, ARG-391
HIF-1α (PDB ID: 1L8C)	Center:(1.1, −5.1, 7.1) Size: (40, 40, 40)	1,4-dicaffeoylquinic-acid	phenylpropanoid	−7.3	4	ALA-102, LEU-101, TRP-74, LYS-75
		Luteolin	flavonoid	−7.2	3	ASP-93, VAL-148, ARG-95
		Compound-**1**	iridoid	−7.1	1	TRP-74
		Verproside	iridoid	−7.1	5	ASP100, SER 36, LYS 21, VAL 148, LEU 88

## Data Availability

The original contributions presented in this study are included in the article/Supplementary Material. Further inquiries can be directed to the corresponding authors.

## Supplementary Materials

The following supporting information can be downloaded at: https://www.mdpi.com/article/10.3390/ph19050678/s1. All supplementary figures are shown in Supplementary File. Identification results of SHZ and SMJ component are shown in Supplementary File S2: Table S1 Identification Results of SHZ Components; Table S2: Identification Results of SMJ Components. All supplementary tables are shown in Supplementary File S3: targets of XZZTP transdermal components in Table S1; disease targets in Table S2; the GO enrichment analysis results in Table S3; the KEGG enrichment analysis results in Table S4; the reference standard information in Table S5. Refs. [46,47,48,49,50,51,52,53,54,55,56,57,58,59,60,61,62] cited in Supplementary Materials.
