# Supplementary material for: The Underlying Pharmacological Mechanisms and Active Components of XZZTP in Modulating Bacterial Inflammation Elucidated by LC-MS/MS, Network Pharmacology, In Vitro Experiments, Molecular Docking, and Dynamics Simulations
Source: Pharmaceuticals (Basel). 2026 Apr 27;19(5):678. doi: 10.3390/ph19050678 (PMC13210147; doi:10.3390/ph19050678)
Supplement: Supplementary file 1 [file pharmaceuticals-19-00678-s001.zip › Supplementary File S1.pdf]

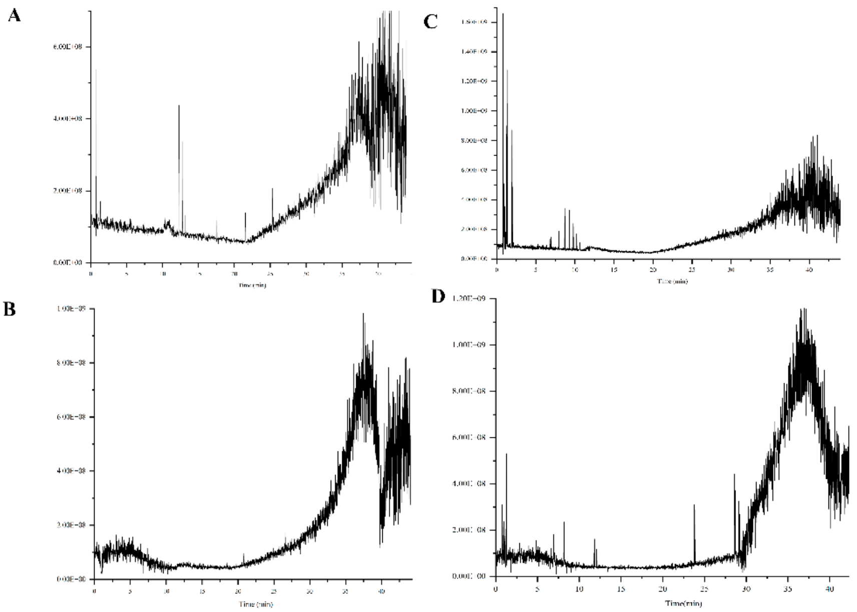

Figure.S1 TICs of XZZTP transdermal sample: Skin sample in positive ion mode (A) and negative ion mode (B); Receptor fluid sample in positive ion mode (C) and negative ion mode (D)

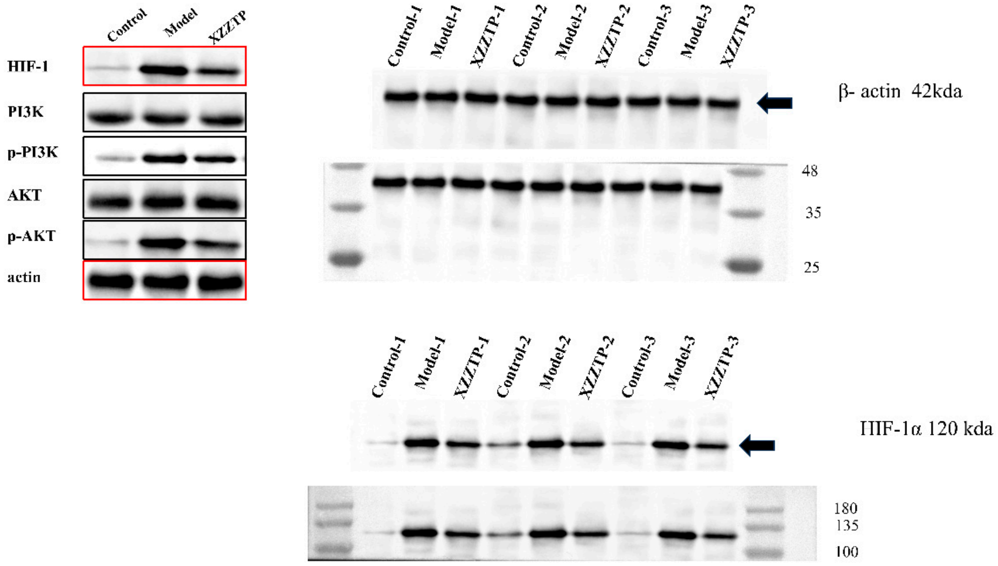

Figure.S2 β-actin and HIF-1α

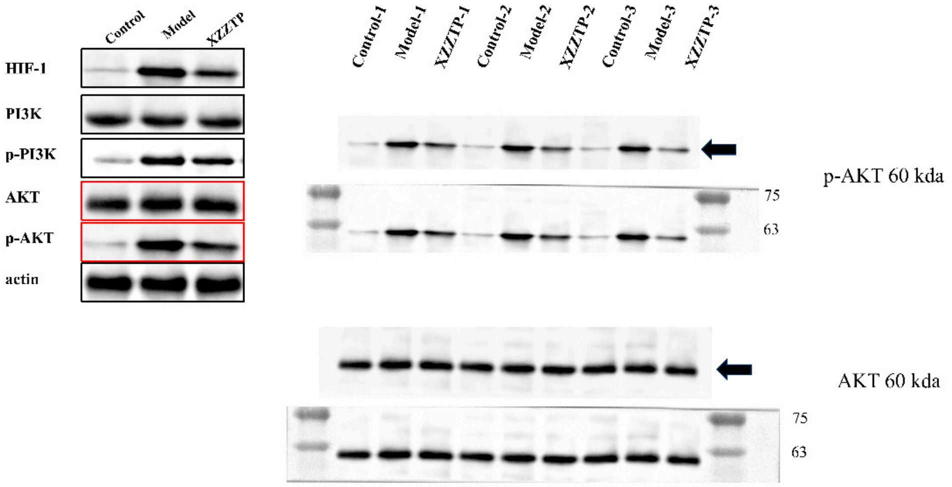

Figure.S3 p-AKT and AKT

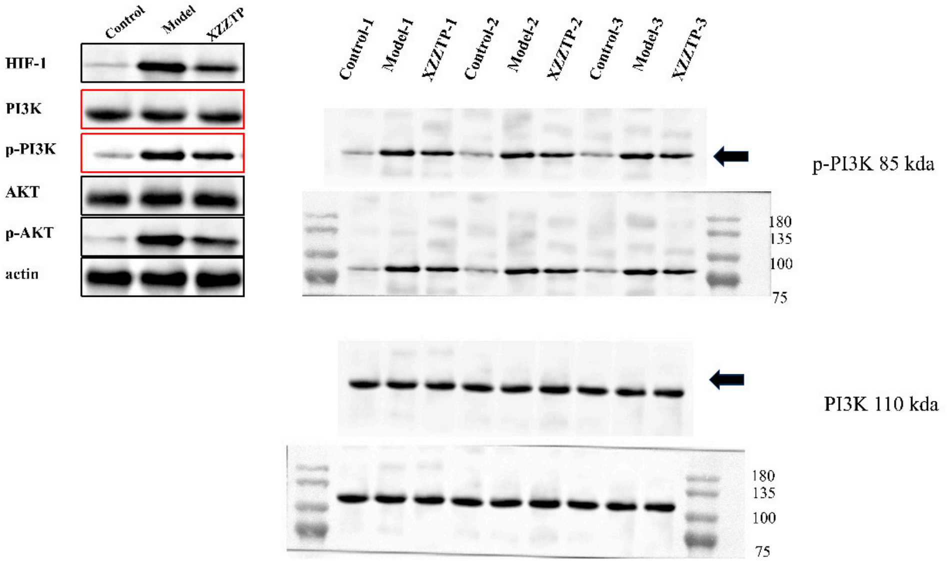

Figure.S4 p-PI3K and PI3K
