# Supplementary material for: The Underlying Pharmacological Mechanisms and Active Components of XZZTP in Modulating Bacterial Inflammation Elucidated by LC-MS/MS, Network Pharmacology, In Vitro Experiments, Molecular Docking, and Dynamics Simulations
Source: Pharmaceuticals (Basel). 2026 Apr 27;19(5):678. doi: 10.3390/ph19050678 (PMC13210147; doi:10.3390/ph19050678)
Supplement: Supplementary file 1 [file pharmaceuticals-19-00678-s001.zip › supplementary File S2.pdf]

Table S1 Identification Results of SHZ Components

No.	Transdermal	Classification	tR（min）	Identification	Molecular Formular	Molecular Weight	Adduct	Calculated *m/z*	Measured *m/z*	MS²
1	√	Phenylpropanoid	2.5	Arteordo coumarin A	$C_{10}H_{10}O_5$	210.0534	$[M+HCOO]^-$	255.0505	255.0496	209.0446, 193.0513, 165.0553, 147.0455
2*	√	Phenylpropanoid	3.71	5-CQA	$C_{16}H_{18}O_9$	354.0945	$[M-H]^-$	353.0873	353.0848	191.0546, 179.0344
3*	√	Phenylpropanoid	3.86	3-CQA	$C_{16}H_{18}O_{10}$	355.0945	$[M-H]^-$	353.0873	353.0850	191.0572, 179.0346
4[1]	√	Amino acid	4.06	tryptophan or isomer	$C_{11}H_{12}N_2O_2$	204.0893	$[M+H]^+$	205.0966	205.0971	188.0698, 146.0598
5		Amino acid	4.79	tryptophan or isomer	$C_{11}H_{12}N_2O_2$	204.0893	$[M+H]^+$	205.0966	205.0971	188.0698, 146.0598
6[2]		Phenylpropanoid	6.12	CQA-glycoside isomer1	$C_{22}H_{28}O_{14}$	516.1485	$[M-H]^-$	515.1406	515.1391	353.0856, 323.0768, 179.0359, 161.0250
7		Phenylpropanoid	6.32	CQA-glycoside isomer2	$C_{22}H_{28}O_{14}$	516.1485	$[M-H]^-$	515.1406	515.1388	353.0856, 323.0768, 179.0359, 161.0250
8[3]		Phenylpropanoid	6.59	caffeic acid-4-glucoside	$C_{15}H_{18}O_9$	342.0956	$[M-H]^-$	341.0884	341.0857	203.0349, 179.0356, 161.0243, 135.0455
9[4]	√	Phenylpropanoid	7.23	6-O-caffeoyl sophorose	$C_{21}H_{28}O_{14}$	504.1474	$[M-H]^-$	503.1406	503.1420	341.0876, 179.0356, 161.0247
10[2]	√	Phenylpropanoid	7.37	3-SQA	$C_{18}H_{22}O_{10}$	398.1207	$[M-H]^-$	397.1140	397.1166	223.0613, 191.0562, 173.0445, 135.0444
11（R）[5]	√	Phenylpropanoid	7.5	caffeic acid	$C_9H_8O_4$	180.0417	$[M-H]^-$	179.0350	179.0358	135.0458
12*	√	Phenylpropanoid	7.56	4-CQA	$C_{16}H_{18}O_9$	357.0945	$[M-H]^-$	353.0873	353.0851	191.0517, 179.0345, 173.0453

Table S1 Identification Results of SHZ Components (Continued)

**No.**	**Transdermal**	**Classification**	**tR（min）**	**Identification**	**Molecular Formular**	**Molecular Weight**	**Adduct**	**Calculated *m/z***	**Measured *m/z***	**MS²**
13		Flavonoid	8.34	quercetin-3,7-di-O-glucoside	$C_{27}H_{30}O_{17}$	626.1478	$[M-H]^-$	625.1410	625.1421	463.0815,301.0372, 300.0296, 283.0236
14[6]	√	Phenylpropanoid	8.62	5-SQA	$C_{18}H_{22}O_{10}$	398.1207	$[M-H]^-$	397.1140	397.1137	223.0613, 191.0552,
15[7]		Phenylpropanoid	8.65	1-CQA	$C_{16}H_{18}O_{8}$	338.1007	$[M-H]^-$	337.0929	337.0939	191.0562, 173.0455,
16	√	Phenylpropanoid	8.79	4-SQA	$C_{18}H_{22}O_{10}$	398.1207	$[M-H]^-$	397.1140	397.1142	223.0602, 191.0554, 179.0352
17*	√	Phenylpropanoid	9.12	1,3-dicaffeoylquinic acid	$C_{25}H_{24}O_{12}$	516.1262	$[M-H]^-$	515.1195	515.1151	353.0908, 335.0780, 191.0562
18*	√	Phenylpropanoid	9.51	3-FQA	$C_{17}H_{20}O_{9}$	368.1102	$[M-H]^-$	367.1035	367.1032	193.0496, 191.0567,
19*	√	Phenylpropanoid	9.84	5-FQA	$C_{17}H_{20}O_{9}$	368.1102	$[M-H]^-$	367.1035	367.1053	193.0504, 191.0554, 173.0460
20*		Flavonoid	10.2	hyperoside	$C_{21}H_{20}O_{12}$	464.0960	$[M-H]^-$	463.0882	463.0912	301.0355, 300.0275, 271.0255, 255.0289,
21	√	Phenylpropanoid	10.26	4-FQA	$C_{17}H_{20}O_{9}$	368.1102	$[M-H]^-$	367.1035	367.1032	193.0490, 191.0567
22*	√	Phenylpropanoid	10.53	ferulic acid	$C_{10}H_{10}O_{4}$	194.0574	$[M-H]^-$	193.0507	193.0499	178.0287, 149.0582, 134.0365
23[2]		Phenylpropanoid	10.83	tricaffeoylquinic acid	$C_{31}H_{34}O_{17}$	678.5839	$[M-H]^-$	677.1723	677.1801	515.1408, 353.0879, 341.0876,335.0764, 191.0562, 179.0349
24		Flavonoid	10.89	kamferol-3-O-Rutoside	$C_{27}H_{30}O_{15}$	594.1579	$[M-H]^-$	593.1512	593.1576	285.0395, 151.0034, 133.0286

Table S1 Identification Results of SHZ Components (Continued)

No.	Transdermal	Classification	tR（min）	Identification	Molecular Formular	Molecular Weight	Adduct	Calculated *m/z*	Mesaured *m/z*	MS²
25*	√	Phenylpropanoid	11.58	3,4-dicaffeoylquinic acid	$C_{25}H_{24}O_{12}$	516.1262	$[M-H]^-$	515.1195	515.1196	353.0879, 335.0774, 299.0589, 255.0672, 203.0349, 191.0561, 179.0349, 173.0459
26[7]		Flavonoid	11.7	3-methoxyluteolin-3-O-rutinoside	$C_{28}H_{32}O_{16}$	624.1685	$[M-H]^-$	623.1618	623.1656	315.0501, 300.0270, 271.0251, 151.0014
27	√	Phenylpropanoid	11.76	3,5-dicaffeoylquinic acid	$C_{25}H_{24}O_{12}$	516.1262	$[M-H]^-$	515.1195	515.1151	353.0908, 191.0555, 179.0353, 135.0455
28*	√	Phenylpropanoid	12.12	4,5-dicaffeoylquinic acid	$C_{25}H_{24}O_{12}$	516.1262	$[M-H]^-$	515.1195	515.1151	191.0555, 179.0359,
29		Phenylpropanoid	12.32	CSQA isomer1	$C_{27}H_{28}O_{13}$	560.1524	$[M-H]^-$	559.1452	559.1450	497.1099, 397.1154, 353.0907, 335.0767
30*	√	Phenylpropanoid	12.46	1,4-dicaffeoylquinic acid	$C_{25}H_{24}O_{12}$	516.1262	$[M-H]^-$	515.1195	515.1151	353.0973, 299.0551, 203.0365, 179.0343, 173.0451, 161.0246
31*	√	Flavonoid	12.58	chrysoeriol-7-O-glucoside	$C_{22}H_{22}O_{11}$	462.1157	$[M-H]^-$	461.1089	461.1071	299.0804, 283.0238, 255.0305, 165.0555
32*	√	Phenylpropanoid	12.66	1,5-dicaffeoylquinic acid	$C_{25}H_{24}O_{12}$	516.1262	$[M-H]^-$	515.1195	515.1151	353.0908, 191.0555, 179.0556, 135.0455
33[2]		Phenylpropanoid	12.85	CFQA isomer1	$C_{26}H_{26}O_{12}$	530.1419	$[M-H]^-$	529.1351	529.1301	353.0888, 367.1009, 179.0337, 161.0250
34	√	Phenylpropanoid	12.87	CSQA isomer2	$C_{27}H_{28}O_{13}$	560.1524	$[M-H]^-$	559.1452	559.1450	497.1099, 397.1154, 353.0907, 335.0767

Table S1 Identification Results of SHZ Components (Continued)

No.	transdermal	Classification	tR（min）	Identification	Molecular Formular	Molecular Weight	Adduct	Calculated *m/z*	Mesaured *m/z*	MS²
35		Phenylpropanoid	13.07	CFQA isomer2	$C_{26}H_{26}O_{12}$	530.1419	$[M-H]^-$	529.1351	529.1351	353.0872, 367.1012, 179.0367
36		Phenylpropanoid	13.26	CFQA isomer3	$C_{26}H_{26}O_{12}$	530.1419	$[M-H]^-$	529.1351	529.1343	353.0851, 367.1013, 179.0344
37		Phenylpropanoid	13.8	CFQA isomer4	$C_{26}H_{26}O_{12}$	530.1419	$[M-H]^-$	529.1351	529.1333	353.0884, 367.1020, 179.0347
38		Phenylpropanoid	13.98	CFQA isomer5	$C_{26}H_{26}O_{12}$	530.1419	$[M-H]^-$	529.1351	529.1404	353.0873, 367.1009, 179.0337
39		Phenylpropanoid	14.23	1,4-diferuloylquinic acid	$C_{27}H_{28}O_{12}$	544.1575	$[M-H]^-$	543.1508	543.1514	367.1066, 349.0920, 193.0502, 173.0444
40*	√	Flavonoid	14.46	luteolin	$C_{15}H_{10}O_{6}$	286.0472	$[M-H]^-$	285.0399	285.0407	257.0452, 241.0508, 199.0400, 175.0395, 151.0037, 133.0294
41[4]		Flavonoid	14.61	5,7,2',4' -tetrahydroxy-5-methoxyflavone	$C_{16}H_{12}O_{7}$	316.0578	$[M-H]^-$	315.0510	315.0506	300.0275, 287.0568, 193.0148, 165.0185
42		Phenylpropanoid	14.79	3,5-diferuloylquinic acid	$C_{27}H_{28}O_{13}$	544.1575	$[M-H]^-$	543.1508	543.1514	367.1028, 349.0917, 193.0505, 173.0439
43	√	Flavonoid	14.83	3,5,3',4'-tetrahydroxy-6,7-dimethoxyflavone	$C_{17}H_{14}O_{8}$	346.0683	$[M+H]^+$	347.0761	347.0756	332.0517, 317.0290
44		Phenylpropanoid	15.03	CFQA isomer6	$C_{26}H_{26}O_{12}$	530.1430	$[M-H]^-$	529.1351	529.1376	367.1036, 353.0879, 193.0506, 173.0455, 135.0452

Table S1 Identification Results of SHZ Components (Continued)

No.	Transdermal	Classification	tR（min）	Identification	Molecular Formular	Molecular Weight	Adduct	Calculated *m/z*	Mesaured *m/z*	MS^2
45		Phenylpropanoid	15.13	4,5diferuloylquinic acid	$C_{27}H_{28}O_{14}$	544.1575	$[M-H]^-$	543.1508	543.1514	367.1032, 349.0947, 193.0513, 173.0453
46	√	Phenylpropanoid	15.36	caffeic acid ethyl ester	$C_{11}H_{12}O_4$	208.0741	$[M-H]^-$	207.0663	207.0661	192.0427, 179.0343, 161.0448
47*	√	Flavonoid	16	apigenin	$C_{15}H_{10}O_5$	270.0523	$[M+H]^+$	271.0601	271.0578	153.0178, 119.0486
48*	√	Flavonoid	16.12	naringenin	$C_{15}H_{12}O_5$	272.0679	$[M-H]^-$	271.0612	271.0613	249.4702, 177.0204, 165.0191, 151.0033,
49*[5]	√	Flavonoid	16.31	hydroxygenkwanin	$C_{16}H_{12}O_6$	300.0628	$[M-H]^-$	299.0556	299.0542	284.032, 227.0350, 212.0482
50*	√	Flavonoid	16.51	chrysoeriol	$C_{16}H_{12}O_6$	300.0628	$[M-H]^-$	299.0556	299.0542	284.032, 256.0375
51[4]		Flavonoid	16.71	5,7,2',4'-tetrahydroxy-6,5'-dimethoxy-flavone	$C_{17}H_{14}O_8$	346.0683	$[M-H]^-$	345.0610	345.0628	330.0406, 315.0176, 287.0187, 243.0302, 180.0057, 164.9823
52*	√	Flavonoid	16.76	jaceosidin	$C_{17}H_{14}O_7$	330.0734	$[M+H]^+$	331.0812	331.0812	316.0595, 301.0343, 273.0371, 245.0441, 186.0150, 168.0050
53		Flavonoid	16.94	3',5,7-trihydroxy-3,4' – dimethoxy-flavone	$C_{17}H_{14}O_7$	330.0734	$[M+H]^+$	331.0812	331.0812	316.0595, 301.0343, 273.0371, 245.0441, 186.0150, 168.0050

Table S1 Identification Results of SHZ Components (Continued)

No.	Transdermal	Classification	tR（min）	Identification	Molecular Formular	Molecular Weight	Adduct	Calculated *m/z*	Mesaured *m/z*	MS²
54 (R) [8]	√	Flavonoid	17.15	5,2',4'-trihydroxy-6,7,5'- trimethoxy-flavone	$C_{18}H_{16}O_8$	360.0840	$[M+H]^+$	361.0918	361.0899	346.0630, 328.0562, 300.0603, 285.0408, 197.0459, 164.0466
55[4]		Flavonoid	17.51	5,7,3'-trihydroxy-6,4',5'-trimethoxy-flavone	$C_{18}H_{16}O_8$	360.0840	$[M+H]^+$	361.0918	361.0899	346.0630, 328.0562, 300.0603, 285.0408, 197.0459, 164.0466
56 (R) [9]	√	Flavonoid	18.39	blumeatin	$C_{16}H_{14}O_6$	302.0785	$[M+H]^+$	303.0863	303.0852	285.0772, 261.0781, 205.0497, 193.0492, 167.0341, 163.0388, 145.0287, 135.0434
57 (R) [8]		Flavonoid	18.8	rhamnetin	$C_{16}H_{12}O_7$	316.0578	$[M-H]^-$	315.0510	315.0506	300.0275, 287.0568, 193.0148, 165.0185
58* (R) [8]		Flavonoid	18.84	3,5-dihydroxy-4',7-dimethoxy-flavone	$C_{17}H_{14}O_6$	314.0785	$[M+H]^+$	315.0863	315.0842	300.0601, 272.0678, 168.0047
59[4]		Flavonoid	19.2	5,4 '-dihydroxy-6,7,3' –trimethoxy-flavone	$C_{18}H_{16}O_7$	344.0891	$[M+H]^+$	345.0969	345.0968	330.0370, 315.0498, 312.0608, 284.0692, 151.0383

Table S1 Identification Results of SHZ Components (Continued)

No.	Transdermal	Classification	tR（min）	Identification	Molecular Formular	Molecular Weight	Adduct	Calculated m/z	Mesaured m/z	MS²
60[4]	√	Flavonoid	19.44	5,2',6'-trihydroxy-6,7,8-trimethoxyflavone	$C_{18}H_{16}O_8$	360.0840	$[M+H]^+$	361.0918	361.0899	346.0630, 328.0562, 300.0603, 285.0408, 197.0459, 164.0466
61（R）[10]	√	Flavonoid	20.7	isosakuranetin	$C_{16}H_{14}O_5$	286.0836	$[M+H]^+$	287.0914	287.0922	269.0795, 245.0804, 161.0592, 153.0178
62*	√	Flavonoid	20.99	acacetin	$C_{16}H_{12}O_5$	284.0679	$[M-H]^-$	283.0606	283.0614	268.0358, 239.0337, 151.0039, 131.7174
63*		Flavonoid	21.16	5,7-dihydroxy-3′,4′-dimethoxyflavone	$C_{17}H_{14}O_6$	314.0785	$[M+H]^+$	315.0863	315.0858	300.0601, 272.0678, 168.0047
64*	√	Flavonoid	21.55	5,7,4 '- trihydroxy-6-methoxyflavone	$C_{16}H_{12}O_6$	300.0628	$[M+H]^+$	301.0707	301.0728	286.0469, 258.0510, 168.0045, 140.0107, 121.0289
65*	√	Flavonoid	25.34	5,7-dihydroxy-3′,4′-dimethoxyflavone	$C_{17}H_{14}O_6$	314.0785	$[M+H]^+$	315.0863	315.0858	300.0601, 272.0678, 168.0047
66（R）[11]	√	Fatty acid	31.58	α-linolenic acid	$C_{18}H_{30}O_2$	278.2247	$[M+H]^+$	279.2320	279.2313	261.2207, 243.2091, 237.1850
67	√	Fatty acid	32.8	oleamide	$C_{18}H_{35}NO$	281.2713	$[M+H]^+$	282.2786	282.2767	265.2525, 247.2415, 177.1641, 163.1478

Note: “(R)” following No. indicates that the component has been reported in literature related to SMJ; “*” denotes that the component was confirmed by comparison with reference standards.

Table S2 Identification Results of SMJ Components

No.	Transdermal	Classification	tR（min）	Identification	Molecular Formular	Molecular Weight	Adduct	Calculated*m*/*z*	Mesaured*m*/*z*	MS²
1*（R）[12]	√	Iridoid	1.28	catalpol or isomer	$C_{15}H_{22}O_{10}$	362.1218	$[M-H]^-$, $[M++HCOO]^-$	407.1200	407.1204	361.1119, 199.0619, 181.0504, 169.0500
2（R）[12]		Iridoid	1.43	catalpol or isomer	$C_{15}H_{22}O_{10}$	362.1218	$[M-H]^-$, $[M+HCOO]^-$	407.1200	407.1204	361.1121, 199.0617, 181.0501, 169.0499
3[13]		Iridoid	1.54	shanzhiside	$C_{16}H_{24}O_{11}$	392.1313	$[M-H]^-$	391.1240	391.1218	345.1175, 229.0733, 183.0671, 123.0459
4（R）[14]		Iridoid	1.91	aucubin	$C_{15}H_{22}O_{9}$	346.1258	$[M-H]^-$, $[M++HCOO]^-$	391.1240	391.1263	345.1227, 183.0674, 165.0565
5[13]		Iridoid	2.33	gardoside	$C_{16}H_{22}O_{10}$	374.1207	$[M-H]^-$	373.1135	373.1142	315.0703, 211.0608, 193.0494, 167.0712,
6		Organic acid	2.76	protocatechuic acid 4-glucoside	$C_{13}H_{16}O_{9}$	316.0800	$[M-H]^-$	315.0722	315.0746	255.0527, 195.0297, 153.0202
7*（R）[14]	√	Iridoid	2.88	geniposidic acid	$C_{16}H_{22}O_{10}$	374.1218	$[M-H]^-$	373.1146	373.1136	353.0944, 211.0600, 167.0708, 149.0600, 123.0455, 119.0213
8*（R）[15]	√	Organic acid	3.1	protocatechuic acid	$C_{7}H_{6}O_{4}$	154.0261	$[M-H]^-$, $[2M-H]^-$	153.0193	153.0189	109.0296
9	√	Iridoid	3.38	compound-1	$C_{22}H_{28}O_{14}$	516.1485	$[M-H]^-$	515.1406	515.1459	153.0187, 225.0408, 353.0888
10		Iridoid	3.56	compound-1 isomer 1	$C_{22}H_{28}O_{14}$	516.1485	$[M-H]^-$	515.1406	515.1422	153.0193, 225.0412, 353.0810

Table S2 Identification Results of SMJ Components (Continued)

No.	Transdermal	Classification	tR（min）	Identification	Molecular Formular	Molecular Weight	Adduct	Calculated *m/z*	Mesaured *m/z*	MS^2
11		Iridoid	3.75	compound-1 isomer 2	$C_{22}H_{28}O_{14}$	516.1485	$[M-H]^-$	515.1406	515.1422	153.0193, 225.0412, 353.0810
12[3]		Phenylpropanoid	4.06	caffeic acid-2-glucoside	$C_{15}H_{18}O_9$	342.0956	$[M-H]^-$	341.0884	341.0857	203.0364, 179.0342, 161.0235, 135.0447
13[3]		Phenylpropanoid	4.25	caffeic acid-3-glucoside	$C_{15}H_{18}O_9$	342.0956	$[M-H]^-$	341.0884	341.0857	203.0349, 179.0349, 161.0243, 135.0452
14[16]		Iridoid	4.96	mussaenosidic acid	$C_{16}H_{24}O_{10}$	376.1364	$[M-H]^-$	375.1291	375.1270	213.0755, 169.0879, 151.0771
15		Organic acid	5.19	vanillic acid 4-β-D-glucopyranoside	$C_{14}H_{18}O_9$	330.0945	$[M-H]^-$	329.0873	329.0880	167.0350
16[3]		Phenylpropanoid	6.29	caffeic acid-1-glucoside	$C_{15}H_{18}O_9$	342.0956	$[M-H]^-$	341.0884	341.0857	221.0442, 179.0342, 161.0237, 135.0447
17		Iridoid	6.75	compound-1 isomer 3	$C_{22}H_{28}O_{14}$	516.1485	$[M-H]^-$	515.1406	515.1453	153.0193, 225.0412, 353.0810
18[3]		Phenylpropanoid	6.98	caffeic acid-4-glucoside	$C_{15}H_{18}O_9$	342.0956	$[M-H]^-$	341.0884	341.0857	221.0447, 179.0346, 161.0238, 135.0459

Table S2 Identification Results of SMJ Components(Continued)

**No.**	**Transdermal**	**Classification**	**tR （min）**	**Identification**	**Molecular Formular**	**Molecular Weight**	**Adduct**	**Calculated *m/z***	**Mesaured *m/z***	**MS²**
19（R） [15]		Organic acid	7.42	vanillic acid	$C_8H_8O_4$	168.0417	$[M-H]^-$	167.0349	167.0349	152.0115, 123.0451, 108.0217
20		Iridoid	7.57	compound-1 isomer 4	$C_{22}H_{28}O_{14}$	516.1485	$[M-H]^-$	515.1406	515.1422	153.0193, 225.0412, 353.0810
21*	√	Phenylpropanoid	7.73	caffeic acid	$C_9H_8O_4$	180.0417	$[M-H]^-$	179.0344	179.0354	135.0452
22		Iridoid	7.75	compound-1 isomer 5	$C_{22}H_{28}O_{14}$	516.1485	$[M-H]^-$	515.1406	515.1422	153.0193, 225.0412, 353.0810
23（R） [17]		Flavonoid	9.68	linariifolioside Ⅱ	$C_{27}H_{30}O_{17}$	626.5152	$[M+H]^+$	627.1555	627.1553	465.0991, 303.0500, 285.0405, 169.0130
24*（R） [14]	√	Iridoid	9.77	verproside or isomer	$C_{22}H_{26}O_{13}$	498.1368	$[M-H]^-$, $[2M-H]^-$	497.1301	497.1292	995.2739, 448.8976, 335.0767, 221.0448, 153.0186
25（R） [14]		Iridoid	9.83	verproside or isomer	$C_{22}H_{26}O_{13}$	498.1368	$[M-H]^-$, $[2M-H]^-$	497.1301	497.1278	335.0771, 221.0459, 153.0197
26*		Flavonoid	10.11	hyperoside	$C_{21}H_{20}O_{12}$	464.0949	$[M-H]^-$	463.0877	463.0912	301.0355, 200.0275, 271.0255, 255.0289, 151.0045

Table S2 Identification Results of SMJ Components(Continued)

No.	Transdermal	Classification	tR（min）	Identification	Molecular Formular	Molecular Weight	Adduct	Calculated *m/z*	Mesaured *m/z*	MS²
27*（R）[15]	√	Phenylpropanoid	10.53	ferulic acid	$C_{10}H_{10}O_4$	194.0585	$[M-H]^-$	193.0507	193.0499	178.0287, 149.0582, 134.0365, 91.0162
28[17]		Flavonoid	10.59	luteolin-7-o-gentiobioside	$C_{27}H_{30}O_{16}$	610.1528	$[M+H]^+$	611.1606	611.1648	449.1108, 287.0540, 241.0503
29*（R）[14]	√	Iridoid	10.81	catalposide	$C_{22}H_{26}O_{12}$	482.1419	$[M-H]^-$	481.1342	481.1404	319.0793, 205.0516, 177.0556
30（R）[18]		Flavonoid	10.99	3′, 4′, 5, 6, 7-pentahydroxyflavone-7-O-6′-O-acetyl-β-D-glucosyl-(1‴→2″)- β-D-glucoside	$C_{29}H_{32}O_{18}$	668.1583	$[M+H]^+$	669.1661	669.1664	507.1159, 447.0898, 303.0508, 285.0367
31（R）[19]		Iridoid	11.14	picroside II or isomer	$C_{23}H_{28}O_{13}$	512.1524	$[M-H]^-$	511.1457	511.1460	349.0905, 235.0629, 167.0359
32（R）[17]	√	Iridoid	11.19	verminoside	$C_{24}H_{28}O_{13}$	524.1524	$[M-H]^-$, $[2M-H]^-$	523.1451	523.1466	361.0939, 179.0345, 161.0237
33*（R）[19]	√	Iridoid	11.51	picroside II or isomer	$C_{23}H_{28}O_{13}$	512.1524	$[M-H]^-$	511.1457	511.1460	349.0912, 235.0619, 167.0353

Table S2 Identification Results of SMJ Components(Continued)

No.	Transdermal	Classification	tR（min）	Identification	Molecular Formular	Molecular Weight	Adduct	Calculated*m/z*	Mesaured *m/z*	MS^2
34[17]		Iridoid	12.32	specioside	$C_{24}H_{28}O_{12}$	508.1575	$[M-H]^-$	507.1503	507.1462	345.0981, 231.0682, 163.047, 145.0282, 119.0513
35（R）[15]		Flavonoid	12.5	apigenin 7-O-b-glucuronide	$C_{21}H_{18}O_{11}$	446.0840	$[M+H]^+$	447.0921	447.0917	373.8639, 271.0579, 153.0175
36*	√	Organic acid	12.54	azelaic acid	$C_9H_{16}O_4$	188.1043	$[M-H]^-$	187.0970	187.0976	125.0972
37（R）[17]	√	Organic acid	12.77	ethyl protocatechuate	$C_9H_{10}O_4$	182.1762	$[M-H]^-$	181.0506	181.0509	153.0195, 109.0296
38（R）[13]		Iridoid	12.79	picroside Ⅲ or isomer	$C_{25}H_{30}O_{13}$	538.1681	$[M-H]^-$	537.1608	537.1630	375.1119, 261.0722, 193.0514, 134.0368
39[13]		Flavonoid	12.8	jaceosidin-7-O- β-D-glucoside	$C_{23}H_{24}O_{12}$	492.1262	$[M+H]^+$	493.1341	493.1369	331.0797, 316.0581, 301.0129, 168.0041, 151.0390, 133.6007
40（R）[13]		Flavonoid	12.95	linariifolioside	$C_{29}H_{32}O_{17}$	652.1634	$[M+H]^+$	653.1712	653.1727	449.1139, 287.0556, 241.0479, 153.0176
41（R）[13]		Iridoid	13.15	picroside Ⅲ or isomer	$C_{25}H_{30}O_{13}$	538.1681	$[M-H]^-$	537.1608	537.1622	375.1123, 261.0719, 193.0517, 134.0359
42		Phenylpropanoid	13.68	caffeic acid ethyl ester or isomer	$C_{11}H_{12}O_4$	208.0741	$[M-H]^-$	207.0663	207.0661	192.0427, 179.0343, 161.0448

Table S2 Identification Results of SMJ Components(Continued)

No.	Transdermal	Classification	tR（min）	Identification	Molecular Formular	Molecular Weight	Adduct	Calculate d*m/z*	Mesaure d*m/z*	MS²
43	√	Phenylethanol glycoside	13.9	martynoside or isomer	$C_{31}H_{40}O_{15}$	652.2362	$[M-H]^-$	651.2294	651.2306	475.1873, 193.0517, 175.0404, 161.0240, 135.0451
44[17]		Iridoid	14.32	veronicoside	$C_{22}H_{26}O_{11}$	466.1470	$[M+HCOO]^-$	511.1466	511.1442	465.1393, 189.0562, 181.0505, 161.0603, 1350447, 121.0292
45*（R）[18]	√	Flavonoid	14.46	luteolin	$C_{15}H_{10}O_6$	286.0472	$[M-H]^-$	285.0399	285.0421	257.0458, 241.0491, 199.0409, 175.0395, 151.0032, 133.0348
46[7]		Phenylethanol glycoside	14.5	martynoside or isomer	$C_{31}H_{40}O_{15}$	652.2362	$[M-H]^-$	651.2294	651.2258	475.1866, 193.0524, 175.0411, 161.0237, 135.0452
47*		Saponin	14.79	protodioscin	$C_{51}H_{84}O_{22}$	1048.5449	$[M+HCOO]^-$	1093.5437	1093.5488	1047.5350, 901.4727, 883.4755, 755.4238
48[20]		Saponin	15.07	Ggraecunin G	$C_{45}H_{72}O_{17}$	884.4764	$[M+H]^+$	885.4842	885.4845	723.4324, 577.3731, 415.3205, 397.3094
49	√	Phenylpropanoid	15.27	Caffeic acid ethyl ester isomer	$C_{11}H_{12}O_4$	208.0741	$[M-H]^-$	207.0663	207.0661	192.0427, 179.0343, 161.0448
50[20]		Saponin	15.9	lucyoside R	$C_{36}H_{58}O_{11}$	666.3974	[M+HCOO]	711.3945	711.3938	665.3920, 503.3372
51*（R）[18]	√	Flavonoid	16.21	apigenin	$C_{15}H_{10}O_5$	270.0523	$[M-H]^-$	269.0455	269.0457	163.0623, 151.0350, 117.0350
52[20]		Saponin	16.51	pennogenin 3-O-β-chacotrioside	$C_{45}H_{72}O_{17}$	884.4764	$[M+H]^+$	885.4842	885.4843	293.1244, 395.2951, 413.3060, 431.3169, 575.3583, 593.3699, 739.4308, 867.4745, 885.4903

Table S2 Identification Results of SMJ Components(Continued)

**No.**	**Transdermal**	**Classification**	**tR（min）**	**Identification**	**Molecular Formular**	**Molecular Weight**	**Adduct**	**Calculate *m*/*z***	**Mesaured*m*/*z***	**MS²**
53[21]	√	Fatty acid	23.59	12,13-dihydroxyoctadec-9-enoic acid	$C_{18}H_{34}O_4$	314.2452	$[M-H]^-$	313.2379	313.2388	295.2280, 277.2176
54[21]	√	Fatty acid	23.68	9,10-epoxy-12（Z）-octadecenoic acid	$C_{18}H_{32}O_3$	296.2357	$[M+H]^+$	297.2424	297.2416	161.1464, 179.1448, 241.1948,
55[21]		Fatty acid	28.09	9(S)-HOTrE	$C_{18}H_{30}O_3$	294.2189	$[M+H]^+$	295.2267	295.2278	135.1171, 147.1173, 149.1326, 151.1118, 161.1332, 179.1434, 241.1948, 259.2054, 277.2165, 295.2267
56[21]		Fatty acid	30.89	parinaric acid	$C_{18}H_{28}O_2$	276.2084	$[M+H]^+$	277.2162	277.2155	135.1226, 147.1295
57*	√	Fatty acid	31.58	α-linolenic acid	$C_{18}H_{30}O_2$	278.2247	$[M+H]^+$	279.2319	279.2311	261.2207, 243.2091, 109.1013
58[21]	√	Fatty acid	32.24	palmitamide	$C_{16}H_{33}NO$	255.2557	$[M+H]^+$	256.2634	256.2622	231.6062, 172.1694
59[22]	√	Fatty acid	37.99	erucylamide	$C_{22}H_{43}NO$	337.3339	$[M+H]^+$	338.3417	338.3409	321.3139, 303.3048, 254.2473

Note: "(R)" following No. indicates that the component has been reported in literature related to SMJ; "*" denotes that the component was confirmed by comparison with reference standards.

Reference

1. Asakawa, D.; Mizuno, H.; Sugiyama, E.; Todoroki, K. Fragmentation Study of Tryptophan-Derived Metabolites Induced by Electrospray Ionization Mass Spectrometry for Highly Sensitive Analysis. *Analyst* **2021**, *146*, 2292–2300, doi:10.1039/D0AN02069A.
2. Ouyang, H.; Li, J.; Wu, B.; Zhang, X.; Li, Y.; Yang, S.; He, M.; Feng, Y. A Robust Platform Based on Ultra-High Performance Liquid Chromatography Quadrupole Time of Flight Tandem Mass Spectrometry with a Two-Step Data Mining Strategy in the Investigation, Classification, and Identification of Chlorogenic Acids in Ainsliaea Fragrans Champ. *J. Chromatogr. A* **2017**, *1502*, 38–50, doi:10.1016/j.chroma.2017.04.051.
3. Zhang, J.-Y.; Zhang, Q.; Li, N.; Wang, Z.-J.; Lu, J.-Q.; Qiao, Y.-J. Diagnostic Fragment-Ion-Based and Extension Strategy Coupled to DFIs Intensity Analysis for Identification of Chlorogenic Acids Isomers in Flos Lonicerae Japonicae by HPLC-ESI-MSn. *Talanta* **2013**, *104*, 1–9, doi:10.1016/j.talanta.2012.11.012.
4. Zhang, P. jie; Cao, Y.; Zhang, K.; Song, yue lin Chemical Profiling of Artemisia Rupestris Using HPLC-IT-TOF-MS. **2020**, *45*.
5. Gao, A. ning Study on the Chemical Components and Biological Activities of Haloxylon Seeds, Shaanxi Normal University, 2017.
6. Colomban, S.; De Rosso, M.; Flamini, R.; Navarini, L. LC–MS Methods Combination for Identification and Quantification of *Trans* -sinapoylquinic Acid Regioisomers in Green Coffee. *J Mass Spectrom* **2023**, *58*, e4970, doi:10.1002/jms.4970.
7. Garran A T Comparative Study of Leonurus Japonicus in Chinese and Western Medicine, China Academy of Chinese Medical Sciences, 2020.
8. Wang, Y. nian Study on the Chemical Composition and Quality of Desert Gah Seeds. Master, Beijing University of Chinese Medicine: Beijing, 2004.
9. Wang, L. yan; Ma, Q.; Bai, jie Comparison of Total Flavonoid and Ainaxanthone Content in Different Parts of

the Desert Gao. *Chinese Journal of Traditional Chinese Medicin* **2015**, *33*, 196–198.

10. Zhang, Y. Screening of Anti-Asthmatic Active Components of Desert Gah Seeds and Their Quality Study. Master, Beijing University of Chinese Medicine: Beijing, 2006.

11. Bai, shouning Overview and Prospects of the Extraction of Artemisia Seeds Oil and Artemisia Gum. *Packaging and Food Machinery* **2000**, *03*, 17–23.

12. zhu, kai xian; yang, dingming; He, yegong Study on the Chemical Constituents of Ranunculus Aquatilis. **1989**, *20*, 647.

13. Wu, X.; Zhou, Y.; Yin, F.; Mao, C.; Li, L.; Cai, B.; Lu, T. Quality Control and Producing Areas Differentiation of Gardeniae Fructus for Eight Bioactive Constituents by HPLC–DAD–ESI/MS. *Phytomedicine* **2014**, *21*, 551–559, doi:10.1016/j.phymed.2013.10.002.

14. Xue, haibing Study on Chemical Constituents and Quantitative Analysis of Five Main Constituents from Pseudolysimachion Linariifolium Subsp. Dilatatum, Shanghai University of Traditional Chinese Medicine, 2020.

15. Hong, jun li; Qin, min jian; Wu, gang Phenolic Components in Water Spinach. *Chinese Natural Medicines* **2008**, *02*, 126–129.

16. Ye, P.; Liang, S.; Wang, X.; Duan, L.; Jiang-Yan, F.; Yang, J.; Zhan, R.; Ma, D. Transcriptome Analysis and Targeted Metabolic Profiling for Pathway Elucidation and Identification of a Geraniol Synthase Involved in Iridoid Biosynthesis from Gardenia Jasminoides. *Industrial Crops and Products* **2019**, *132*, 48–58, doi:10.1016/j.indcrop.2019.02.002.

17. Xue, H. Study on the Chemical Components of Medicinal Water Radish and Quantitative Analysis of Five Main Components, Shanghai University of Traditional Chinese Medicine, 2020.

18. Ma, cuiying Study on Flavonoid Compounds in Hydrocotyle Vulgaris. *Acta Pharmaceutica Sinica* **1991**, *03*, 203–208.

19. Zhao, X.; Wei, J.; Yang, M. Simultaneous Analysis of Iridoid Glycosides and Anthraquinones in Morinda Officinalis Using UPLC-QqQ-MS/MS and UPLC-Q/TOF-MSE. Molecules 2018, 23, 1070. https://doi.org/10.3390/molecules23051070.

20. Ge, Y.-W.; Zhu, S.; Yoshimatsu, K.; Komatsu, K. MS/MS Similarity Networking Accelerated Target Profiling of Triterpene Saponins in Eleutherococcus Senticosus Leaves. *Food Chemistry* **2017**, *227*, 444–452, doi:10.1016/j.foodchem.2017.01.119.

21. Zaher, A.M.; Moharram, A.M.; Davis, R.; Panizzi, P.; Makboul, M.A.; Calderón, A.I. Characterisation of the Metabolites of an Antibacterial Endophyte Botryodiplodia Theobromae Pat. of Dracaena Draco L. by LC–MS/MS. Nat. Prod. Res. 2015, 29, 2275–2281. https://doi.org/10.1080/14786419.2015.1012715.

22. Narayana, R.; Mohana, C.; Kumar, A. Analytical Characterization of Erucamide Degradants by Mass Spectrometry. *Polymer Degradation and Stability* **2022**, *200*, 109956, doi:10.1016/j.polymdegradstab.2022.109956.
